# Role of Strontium Cations in ZSM-5 Zeolite in the Methanol-to-Hydrocarbons Reaction

**DOI:** 10.1021/acs.jpclett.3c01259

**Published:** 2023-07-13

**Authors:** Anna Liutkova, Victor Drozhzhin, Jason M. J. J. Heinrichs, Valentin Jestl, Angelina Evtushkova, Brahim Mezari, Álvaro Mayoral, Nikolay Kosinov, Emiel J. M. Hensen

**Affiliations:** † Laboratory of Inorganic Materials and Catalysis, Department of Chemical Engineering and Chemistry, Eindhoven University of Technology, P.O. Box 513, 5600 MB Eindhoven, The Netherlands; ‡ Instituto de Nanociencia y Materiales de Aragón (INMA), CSIC-Universidad de Zaragoza, 50009 Zaragoza, Spain; § Laboratorio de Microscopías Avanzadas (LMA), Universidad de Zaragoza, 50018 Zaragoza, Spain

## Abstract

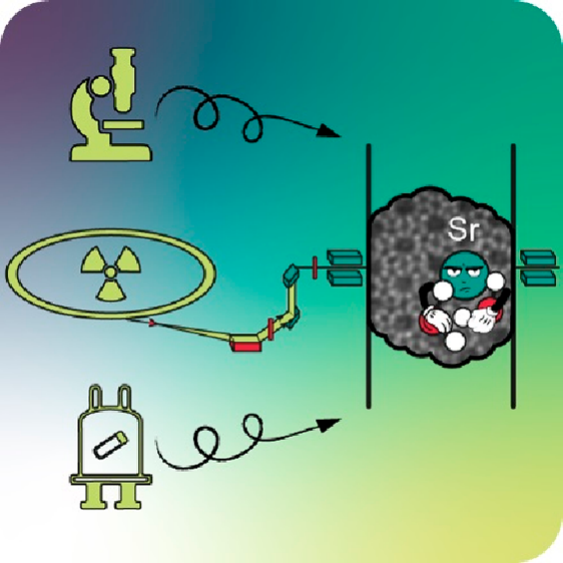

The selectivity of the methanol-to-hydrocarbons (MTH) reaction can be tuned by modifying zeolite catalysts with alkaline earth metals, which typically increase propylene selectivity and catalyst stability. Here we employed Sr^2+^ as its higher atomic number in comparison to the zeolite T atoms facilitates characterization by scanning transmission electron microscopy and operando X-ray absorption spectroscopy. Sr^2+^ dispersed in the ZSM-5 micropores coordinates water, methanol, and dimethyl ether during the MTH reaction. Complementary characterization with nuclear magnetic resonance spectroscopy, thermogravimetric analysis combined with mass spectrometry, operando infrared spectroscopy, and X-ray diffraction points to the retention of substantially more adsorbates during the MTH reaction in comparison to Sr-free zeolites. Our findings support the notion that alkaline earth metals modify the porous reaction environment such that the olefin cycle is favored over the aromatic cycle in the MTH, explaining the increased propylene yield and lower deactivation rate.

Methanol-to-hydrocarbons (MTH) is an industrially relevant reaction to obtain valuable light olefins such as propylene.^1^ The MTH reaction is catalyzed by zeolites^2^ and it can be described using the concept of a dual cycle hydrocarbon pool mechanism.^3^ The production of propylene and higher olefins on one hand and ethylene and aromatics on the other hand are linked to two different types of zeolite-occluded reaction intermediates. By modifying the zeolite, the relative contribution of the two types of intermediates can be changed, thus allowing some control over the product distribution.^4^ Previously, it has been found that the propylene selectivity can be increased by promoting ZSM-5 zeolite with alkaline earth cations.^5^ The origin of this promotion has been linked to destabilization of aromatic precursors.^6−8^ In a previous work, we investigated the evolution of the hydrocarbon pool components in HZSM-5, Na/ZSM-5, and Ca/ZSM-5 catalysts.^9^ We found that Ca/ZSM-5 retains a much higher amount of adsorbates such as water, methanol, and dimethyl ether (DME), which decreases the formation of bulky aromatic hydrocarbon pool species. Recently, it was demonstrated that Sr modification of ZSM-5 also increases the selectivity toward propylene and extends the lifetime of the catalyst in MTH reaction.^10^ Thus, it is likely that the mechanism underlying promotion by Sr is the same as that earlier observed for Ca, implying an important role of retention of reaction intermediates. While typically Mg^2+^ and Ca^2+^ are used to modify MTH catalysts, here we employed Sr^2+^ as its higher atomic number in comparison to the zeolite T atoms facilitates characterization by transmission electron microscopy and X-ray absorption spectroscopy.

In the present study, we confirmed that Sr modification impacts the catalytic performance of ZSM-5 zeolite in the MTH reaction in the same way as other earth alkaline cations. We employed a range of operando and quasi-in-situ techniques—thermogravimetric analysis combined with mass spectrometry (TGA-MS), solid-state magic angle spinning nuclear magnetic resonance spectroscopy (NMR), infrared spectroscopy (IR), and X-ray diffraction (XRD)—to investigate the location and coordination environment of Sr and the nature of the intrazeolitic reaction intermediates. Operando X-ray absorption spectroscopy (XAS) study combined with scanning transmission electron microscopy (STEM) demonstrated the presence of highly dispersed Sr species over working ZSM-5 catalysts, which can coordinate water, methanol, and DME molecules during the MTH reaction.

A total of 5 zeolite catalysts were synthesized, namely, HZSM-5, 0.1Sr/ZSM-5, 0.2Sr-ZSM-5, 0.4Sr/ZSM-5, and Na/ZSM-5. The proton form HZSM-5 was obtained by calcining a commercial NH_4_ZSM-5 powder (Alfa Aesar) at 550 °C for 5 h. *x*Sr/ZSM-5 catalysts (where *x* is the Sr content, i.e., 0.11, 0.22, and 0.44 mmol g^–1^) were prepared by incipient wetness impregnation of the calcined zeolites in their proton form with aqueous solutions of Sr(NO_3_)_2_ (Alfa Aesar, 99.0%), following a procedure described elsewhere.^11^ The targeted Sr contents were 1, 2, and 4 wt %. For the preparation of ion-exchanged Na/ZSM-5, we followed a procedure described in our previous work.^9^ Further details about catalyst preparation are provided in the Supporting Information.

The most important physicochemical properties of HZSM-5, 0.1Sr/ZSM-5, 0.2Sr/ZSM-5, 0.4Sr/ZSM-5, and Na/ZSM-5 catalysts are provided in Table 1. The micropore volumes of the 5 samples are close to 0.14 cm^3^ g^–1^. Bro̷nsted acid sites (BAS) and Lewis acid sites (LAS) were quantitatively characterized by IR spectroscopy of adsorbed pyridine (Figure S3 and Table 1).^12^ The concentration of BAS as probed by pyridine IR and ^1^H NMR spectroscopy decreases in the order HZSM-5 ≫ Na/ZSM-5 ≈ 0.1Sr/ZSM-5 > 0.2Sr/ZSM-5 > 0.4Sr/ZSM-5. This decrease upon modification of ZSM-5 with Na and Sr goes together with an increased number of LAS. The M/Al atomic ratios of the metal-modified HZSM-5 zeolites were in the range of 0.2–0.8 for M = Sr and 0.3 for M = Na.

**Table 1:** Physicochemical Properties of Zeolite Catalysts

catalyst	*S*_total_ (m^2^ g^–1^)	*S*_micro_ (m^2^ g^–1^)	*S*_external_ (m^2^ g^–1^)	*V*_micro_ (cm^3^ g^–1^)	Si/Al^a^	FAl^b^ (%)	BAS (μmol g^–1^), ^1^H NMR	M/Al ratio^a^	exchange degree^c^ (%)	BAS/LAS (μmol g^–1^)^d^
HZSM-5	334	315	20	0.14	33	93.5	428	n.a.	n.a.	501/69
0.1Sr/ZSM-5	329	307	23	0.14	35	94.0	317	0.22	26	260/238
0.2Sr/ZSM-5	336	315	22	0.14	35	97.0	297	0.40	31	165/318
0.4Sr/ZSM-5	315	296	19	0.13	35	99.0	137	0.84	68	118/370
Na/ZSM-5	322	302	20	0.14	36	93.4	264	0.33	38	268/226

a Measured by ICP elemental analysis.

b Fraction of framework Al as determined by ^27^Al MAS NMR.

c Determined by the fractional occupation of initial BAS by the metal ions as probed by ^1^H NMR spectroscopy.

d IR spectroscopy of adsorbed pyridine.

As Sr is significantly heavier than the zeolite T atoms, STEM can be used to image the Sr atoms in the Sr-modified ZSM-5 zeolite. C_s_-corrected STEM coupled with ADF and ABF detectors has been employed before to image metals in zeolite frameworks with atomic resolution, where limiting the electron dose can avoid significant damage of the zeolite by the electron beam.^13−16^ Here, we used C_s_-corrected STEM to study the location of Sr by comparing HZSM-5 and 0.4Sr/ZSM-5 (Figure 1). We chose the sample with the highest Sr content to probe the possible agglomeration of Sr species. The used 0.4Sr/ZSM-5 sample was obtained after 1 h on a methanol stream at a reaction temperature of 450 °C.

**Figure 1:**
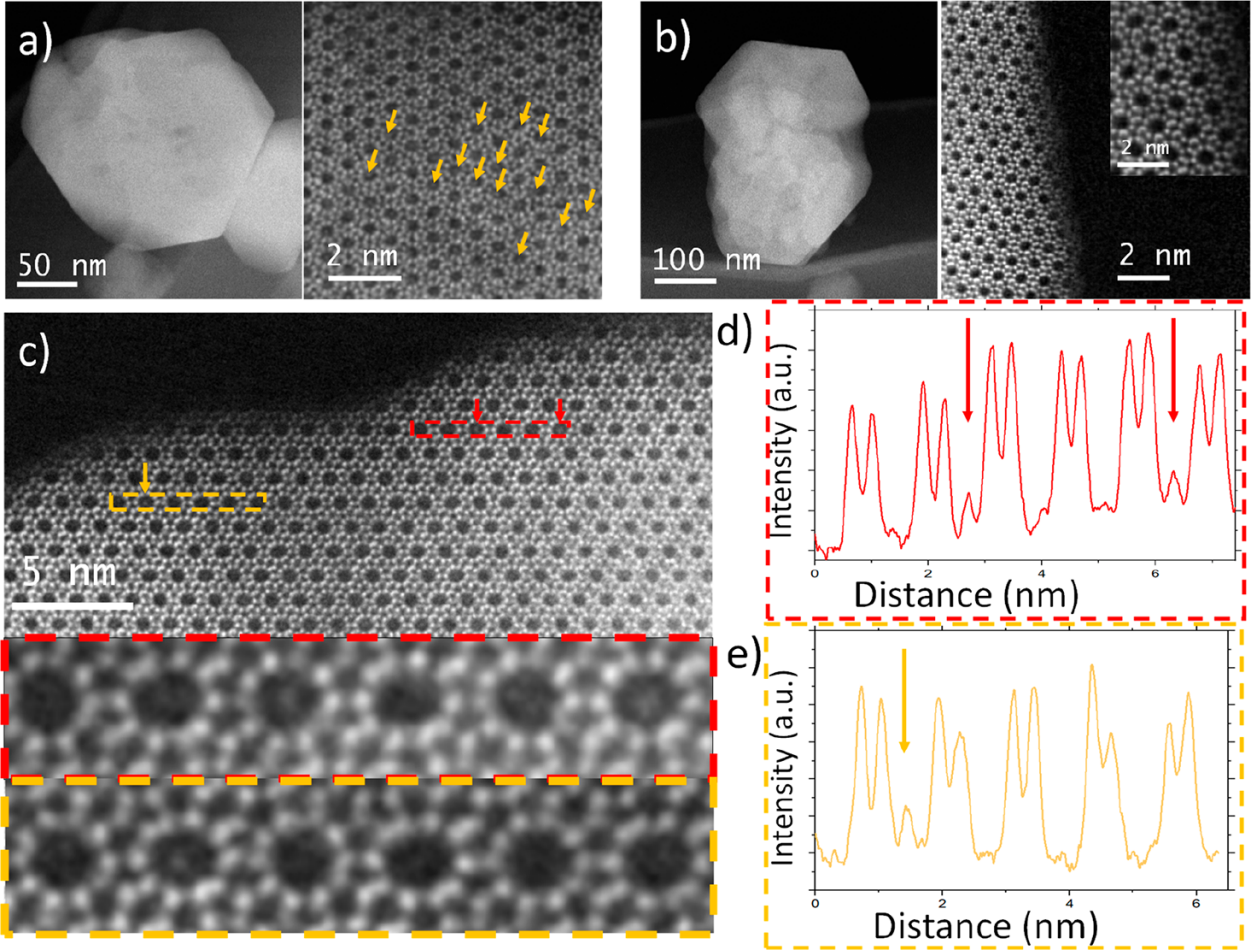
C_s_-corrected STEM-ADF images of used 0.4Sr/ZSM-5 catalyst (a, c) and (b) fresh HZSM-5 zeolite. (d, e) Intensity profile along the dashed rectangle for 0.4Sr/ZSM-5 catalyst; arrows correspond to Sr moieties retained in zeolite. Conditions: C_s_-corrected STEM coupled with ADF and ABF image modes at 300 kV. The used sample represents 0.4Sr/ZSM-5 after reaction for 1 h on methanol stream, 450 °C, 25 mg of catalyst, 12 kPa of MeOH, carrier, 30 mL min^–1^ He, WHSV 12 h^–1^.

From the inspection of the edge of the crystals, we did not observe Sr-containing clusters or nanoparticles on the zeolite external surface (see low-magnification images displayed in Figure 1a,b). Intracrystalline Sr species were imaged along the *b*-axis, which represents the direction along the straight channels and intersections with zigzag channels. Additional HZSM-5 data are presented in Figure S7, where the atomic-resolution images of three different crystals are shown. The absence of signals in the 10MRs viewed along the *b*-axis in the C_s_-corrected STEM ADF images (Figure S7a,b) shows that the pores are empty. Furthermore, Figure S7c shows a C_s_-corrected STEM ABF image taken along the *a*-axis, which corroborates that the sinusoidal pores are also empty (Figure 1b). Differently, the images of the used 0.4Sr/ZSM-5 sample along the *b*-axis clearly show that many of the straight channels are occupied (see arrows in Figures 1a and S8). This can also be appreciated from the increased intensity inside some of the 10MR pores, as opposed to the intensity in empty pores indicated in Figure 1c–e. Considering that these images are projections of several columns, we cannot determine whether the signal in occupied pores is due to one or several atoms, although the latter is more likely. Within the limits of the method, we do not see a preferential location for Sr in the channels. Most of the signals are off-centered, although some of the Sr atoms were also observed in the center of the channels and even in the 6MR pores as can be seen for instance in Figure S8b. Energy-dispersive X-ray spectroscopy of several regions confirms the presence of Sr in these images (Figure S8d).

Overall, STEM shows that Sr is highly dispersed in the micropores of the used 0.4Sr/ZSM-5 catalyst. This is in line with the notion that the Sr cations are present at cation-exchange positions of the zeolite after preparation,^17^ as also follows from the acidity characterization. The presence of relatively large Sr cations in the zeolite micropores will modify the local pore environment, which can influence the catalytic events, taking place in the confined space of the ZSM-5 pores, during the methanol conversion.

The performance of the calcined materials in the MTH reaction at 450 °C is presented in Figure 2a,b. Compared to HZSM-5, the propylene selectivity, overall catalyst stability, and, consistent with this, the amount of methanol converted per Bro̷nsted acid site are significantly higher for 0.1Sr/ZSM-5 and 0.2Sr/ZSM-5. The selectivity toward coke was the lowest for 0.2Sr/ZSM-5 (Figure S9). The 0.4Sr/ZSM-5 sample with the highest Sr loading (i.e., more than 1 Sr^2+^ per 2 Al sites) shows a much worse performance, with a low initial methanol conversion. These findings demonstrate the strong impact of the presence of Sr cations replacing BAS on the catalytic performance. A control experiment with Na/ZSM-5, which contains the same amount of remaining BAS as 0.1Sr/ZSM-5, shows that Na modification does not lead to significant promotion of the MTH reaction. This confirms the positive effect of Sr modification of ZSM-5 in the MTH reaction in terms of increased selectivity toward C_3+_ olefins and a prolonged catalytic lifetime.^10^

**Figure 2:**
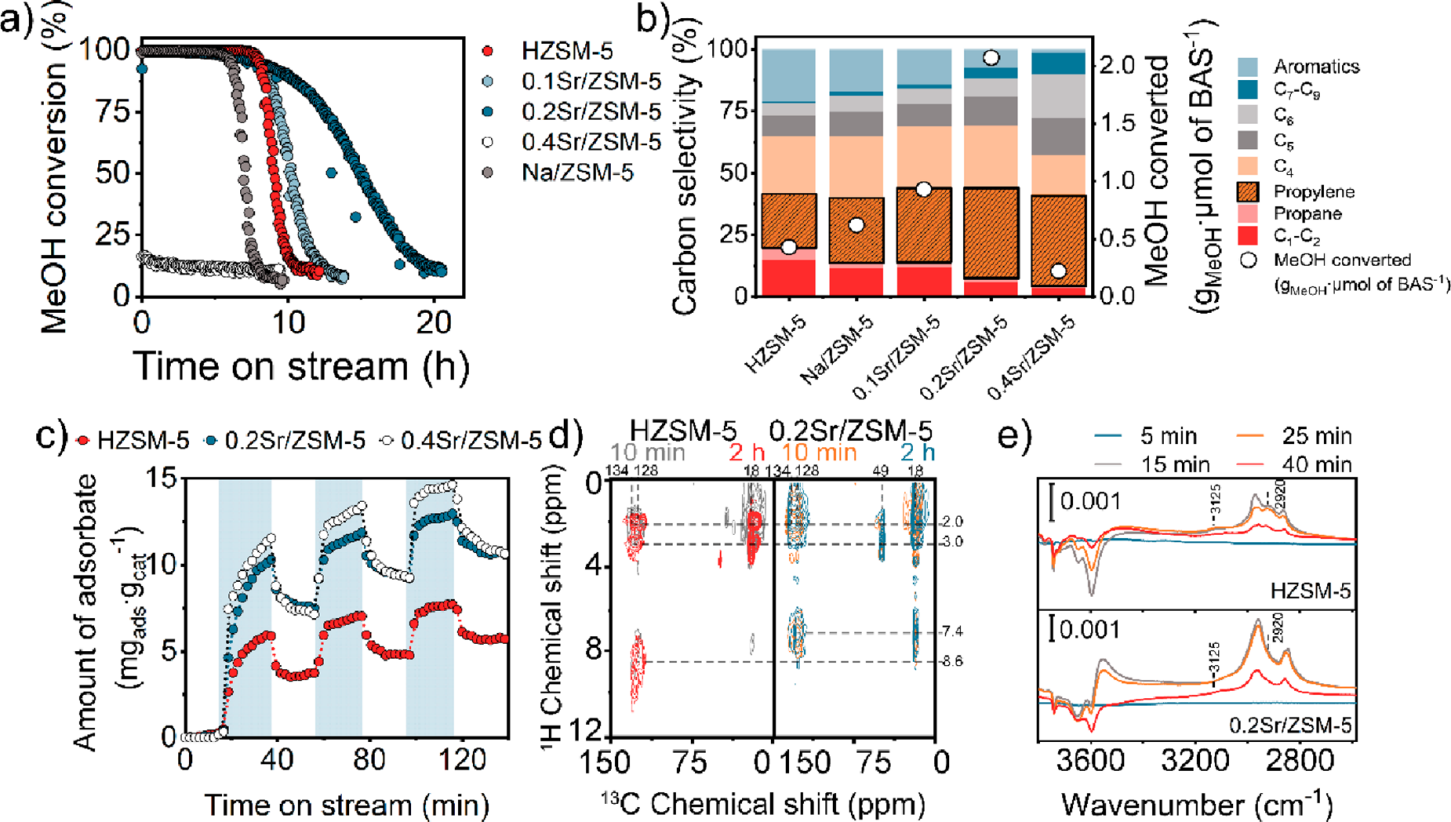
(a) Conversion of methanol as a function of time on stream for the various ZSM-5 catalysts. (b) Overall carbon selectivity during the MTH reaction after 1 h time on stream (TOS) and the amount of converted methanol normalized by the amount of BAS. Conditions: 450 °C, 25 mg of catalyst, 12 kPa of methanol, carrier, 30 mL min^–1^ He, WHSV 12 h^–1^. (c) Operando TGA measurements obtained in the presence (blue highlight) and absence of the methanol feed over 0.2Sr/ZSM-5, 0.4Sr/ZSM-5, and HZSM-5. Conditions: 350 °C, 10 mg of catalyst, carrier, 80 mL min^–1^ He, 0.8 kPa of MeOH. (d) ^1^H–^13^C {^1^H} HETCOR MAS NMR 2D spectra of used HZSM-5 (left) and 0.2Sr/ZSM-5 (right) catalysts after 10 min and 2 h on stream. Conditions: 350 °C, 100 mg of catalyst, carrier: 30 mL min^–1^ He, 12 kPa of ^13^C MeOH. (e) Operando IR measurements of 0.2Sr/ZSM-5 catalyst in the presence (0–20 min TOS) and absence (20–40 min TOS) of the methanol feed and the corresponding Δabs spectra: OH region and C–H region. The Δabs spectra were obtained by subtraction of the first spectrum recorded at 350 °C in the absence of methanol from all other spectra. Conditions: 350 °C, 15 mg of catalyst pellet, 0.12 kPa of methanol, carrier 130 mL min^–1^ He.

Using operando TGA-MS to monitor the mass of the catalyst during switches between CH_3_OH + He and dry He, we determined the amount of species formed and retained on the catalysts (Figure 2c). The optimum 0.2Sr/ZSM-5 catalyst retains a significantly higher amount of adsorbates, hydrocarbons, oxygenates, and water at a similar conversion level as compared to the parent HZSM-5 (Figure S10).^9^^1^H–^13^C NMR spectra of 0.2Sr/ZSM-5 obtained after 10 min and 2 h of MTH reaction in ^13^CH_3_OH (Figure 2d) show aliphatic and methoxy species (signals at 15–25 ppm ^13^C, 0–4 ppm ^1^H, 50 ppm ^13^C, and 2 ppm ^1^H, Figure 2d).^9^ Operando IR demonstrates that 0.2Sr/ZSM-5 retains a significant amount of water and methanol/DME upon switching from CH_3_OH + He to a dry He flow (3600–3200 and ∼2900 cm^–1^ regions, Figure 2e).^18^ The IR measurements of the parent HZSM-5 zeolite indicate much lower amounts of methoxy and water. This clearly shows that the Sr species enhance the interaction of the catalyst with methanol, water, and other adsorbates (Figures S11 and S12). For HZSM-5, the presence of (methylated) benzenes is evident from the IR bands at 2920 and 3125 cm^–1^,^19,20^ which is in line with NMR characterization of the used HZSM-5 catalyst. Operando XRD was used to follow changes in the unit cell of the zeolites upon interaction with adsorbates (Figure S13).^21−23^ These measurements recorded during the MTH reaction show a more significant expansion of the unit cell of 0.2Sr/ZSM-5 in comparison to HZSM-5. The expansion is mostly irreversible, which is most likely due to the retention of some adsorbates, as found by TGA-MS.

The structure of the dispersed Sr species during the MTH reaction was followed using operando XAS at the Sr K-edge for the optimal 0.2Sr/ZSM-5 catalyst upon switches between different gas flows (CH_3_OH/He → He and water/He → He) at 4 temperatures (50, 200, 350, and 450 °C). Prior to the operando experiments, it was verified that the EXAFS spectra of 0.2Sr/ZSM-5 and 0.4Sr/ZSM-5 are comparable (Figure S14). Next, we collected Sr K-edge (16105 eV) XANES and EXAFS spectra of the 0.2Sr/ZSM-5 catalyst during the conversion/adsorption of methanol, while the effluent gas was analyzed with MS. The setup of these experiments is shown in Figure S5a.

First, we analyzed the switch from CH_3_OH/He → He at a reaction temperature of 450 °C (Figure 3a) using a combined XANES-MS analysis (Figure S15d). As shown by Figure S15d, we observed a high conversion of methanol (low *m*/*z* = 31 signal) and formation of propylene (intense *m*/*z* = 41 signal). The observation of the propylene signal shows that the catalyst was active in the MTH reaction in these experiments and that a functional hydrocarbon pool was developed in the zeolite pores. The operando Sr K-edge XANES show changes in the intensity of the white line feature with time on MeOH stream (Figure 3a). As unequivocal assignment of the spectral features in the XANES to reference compounds is not possible (Figure S17a), we prepared a reference sample by exposing the 0.2Sr/ZSM-5 to water vapor (2.2 kPa) at 450 °C. The intensity of the white line was higher for the hydrated state of Sr moieties as compared to the dry conditions. Accordingly, we speculate that the coordination of adsorbates causes an increase in white line intensity.

**Figure 3:**
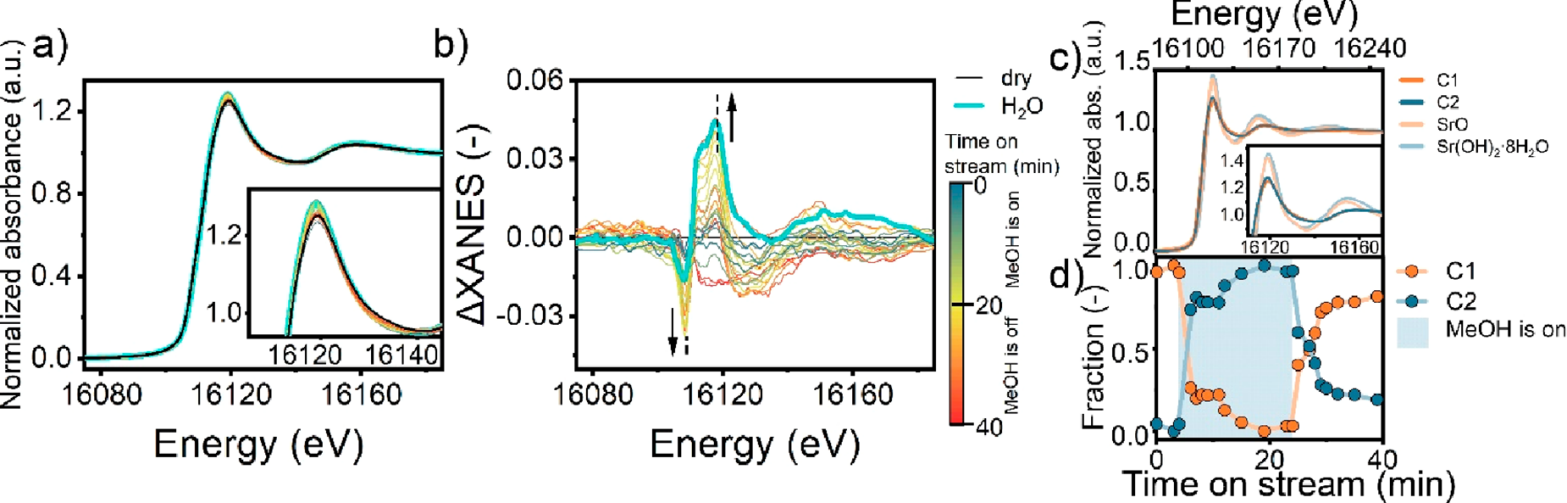
(a) Operando XANES measurements performed over 0.2Sr/ZSM-5 catalyst in presence (0–20 min) and absence of the methanol feed (20–40 min). (b) Corresponding ΔXANES spectra. A separate experiment with water was used as a reference (cyan line). (c) Pure component spectra C1 and C2 compared to reference compounds SrO and Sr(OH)_2_·8H_2_O. (d) Fractions of spectral components determined by MCR-ALS analysis of operando XANES measurements over 0.2Sr/ZSM-5 catalyst in presence and absence of the methanol feed. Conditions: 450 °C, 25 mg of catalyst, 2.2 kPa of methanol, carrier, 30 mL He min^–1^.

In order to emphasize small differences in the XANES, difference XANES (ΔXANES) spectra are presented in Figure 3b obtained by subtraction of the first spectrum recorded for the dried zeolite from all other spectra. We observed that the ΔXANES features at 16110 and 16120 eV became more intense upon exposure to the methanol feel, reaching after 20 min the same intensity as the intensity for the hydrated zeolite (Figure 3a,b). When the feed was switched to a He flow, the intensity of these features decreased again. The shift of the edge position of ∼2–3 eV to higher energies (ΔXANES feature at 16110 eV) provided another indication for changes in the hydration state of Sr^2+^.^24^ Multivariate curve resolution-alternating least-squares (MCR-ALS) analysis showed that the operando XANES can be best described by two components (Figure 3c,d). Details of MCR-ALS analysis are provided in Figures S18 and S19.^22,25^ Component C1, which is similar to the XANES spectrum of the SrO reference, can be used to describe the spectrum of 0.2Sr/ZSM-5 in the absence of methanol. During methanol conversion, adsorption of water, methanol, and DME, and possibly, hydrocarbons, on Sr^2+^ species leads to the appearance of a second spectral component C2, which resembles the Sr(OH)_2_·8H_2_O reference. The component spectra and corresponding reference spectra are shown in Figure 3c. In line with the TGA-MS and IR findings, the adsorbates in the zeolite pores cannot be fully removed after the switch to He, and as much as 25% of the C2 component is preserved after flowing dry He for 20 min (Figure 3d).

Analysis of ΔXANES spectra obtained for switches from CH_3_OH/He → He at other temperatures demonstrates that the ΔXANES features mostly resemble those obtained during water cofeeding (Figure 4c,d). Although the adsorbates are removed faster from the Sr centers at higher reaction temperatures (350 and 450 °C, Figure 4g,h), they are not completely removed after 20 min in dry He. For lower temperatures (50 and 200 °C), where no MTH reaction but adsorption and dehydration of methanol and possible formation of methoxy groups can take place, the intensity of these ΔXANES features is mostly preserved after the switch to pure He (Figure 4a,b,e,f). Overall, this analysis of difference spectra demonstrates that Sr cations in ZSM-5 catalyst interact with adsorbates and that even though the coverage will decrease with increasing temperature, there still remain adsorbates at 450 °C. This flexibility in Sr coordination was previously demonstrated with XANES analysis of aqueous Sr compounds by D’Angelo et al.^24^

**Figure 4:**
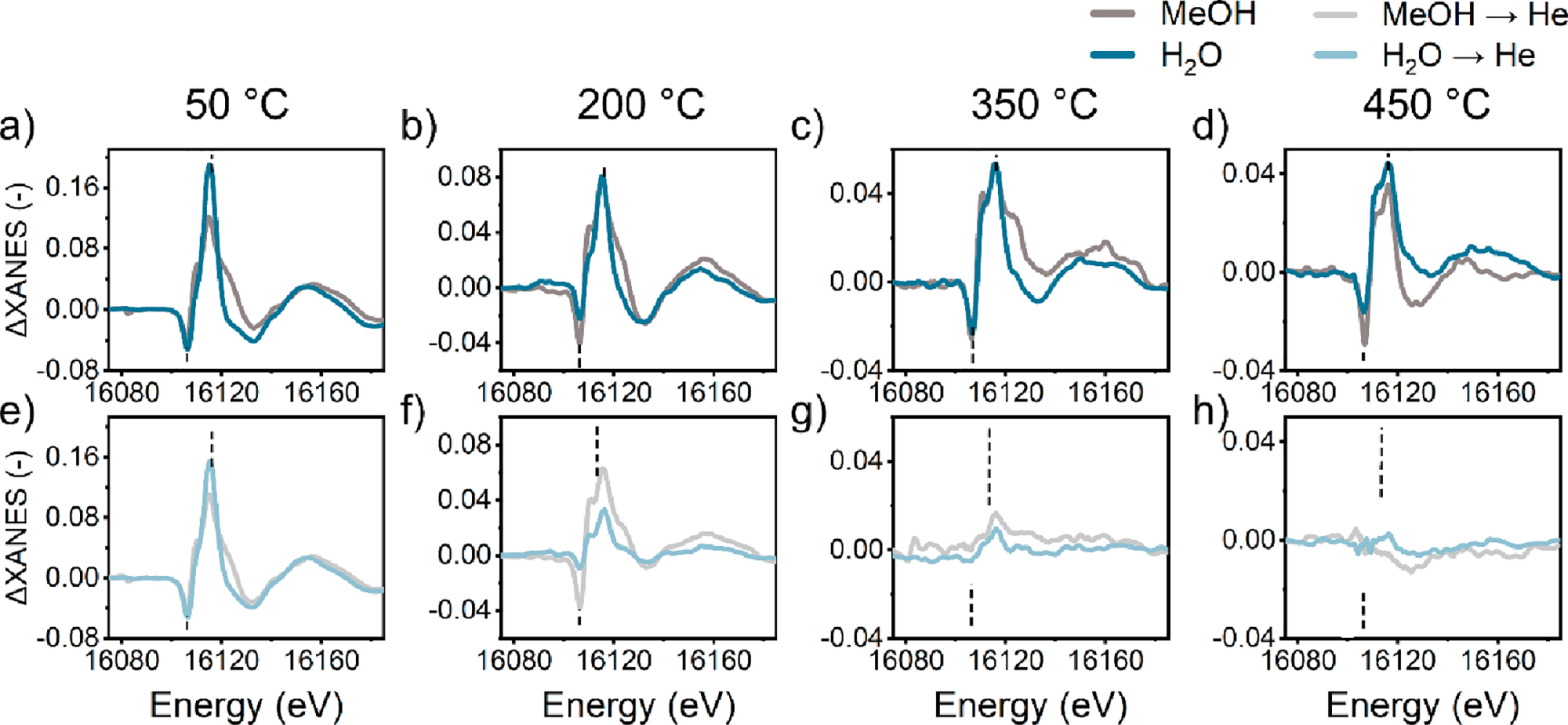
ΔXANES spectra of 0.2Sr/ZSM-5 catalyst recorded in presence and absence of different molecules in the feed. ΔXANES difference is obtained by subtraction of spectrum of dry 0.2Sr/ZSM-5 from intermediate XANES spectra exemplified in Figure S16. Conditions: (a, e) 50 °C, (b, f) 200 °C, (c, g) 350 °C, (d, h) 450 °C, 25 mg of catalyst, 2.2 kPa of feed (methanol or water), carrier, 30 mL He min^–1^.

To examine the coordination number of Sr in the 0.2Sr/ZSM-5 catalyst, we performed EXAFS measurements at 50 °C during CH_3_OH/He → He and water/He → He switches (Figure 5). Only the first coordination shell was fitted (Table S1). The EXAFS spectra and the corresponding fits are shown in Figures S20 and S21. Compared to the Sr–O coordination number of 8 in cubic SrO, the Sr atoms in the as-prepared 0.2Sr/ZSM-5 coordinate ∼6 O atoms. Upon dehydration of the catalyst in He flow at elevated temperature and subsequent cooling to 50 °C, the Sr–O coordination number decreases to ∼4 (0.2Sr/ZSM-5 dry in Figure 5a). After introduction of water in the flow, the coordination number increases again to ∼6. Upon a switch from CH_3_OH/He → He, the coordination number of Sr increases to ∼5 in the presence of MeOH as compared to the dehydrated state. The lower coordination number observed in the presence of methanol as compared to water adsorption can be explained by the larger kinetic diameter of methanol compared to water (3.6 vs 2.7 Å) because all other parameters of the step-response experiments are the same (partial pressure of substrates, flows, temperature). Both in step-response experiments with water and methanol, the coordination number remained high after switching to a dry He feed, which corresponds well with the XANES experiments (Figure 4a,e) and the other results discussed above.

**Figure 5:**
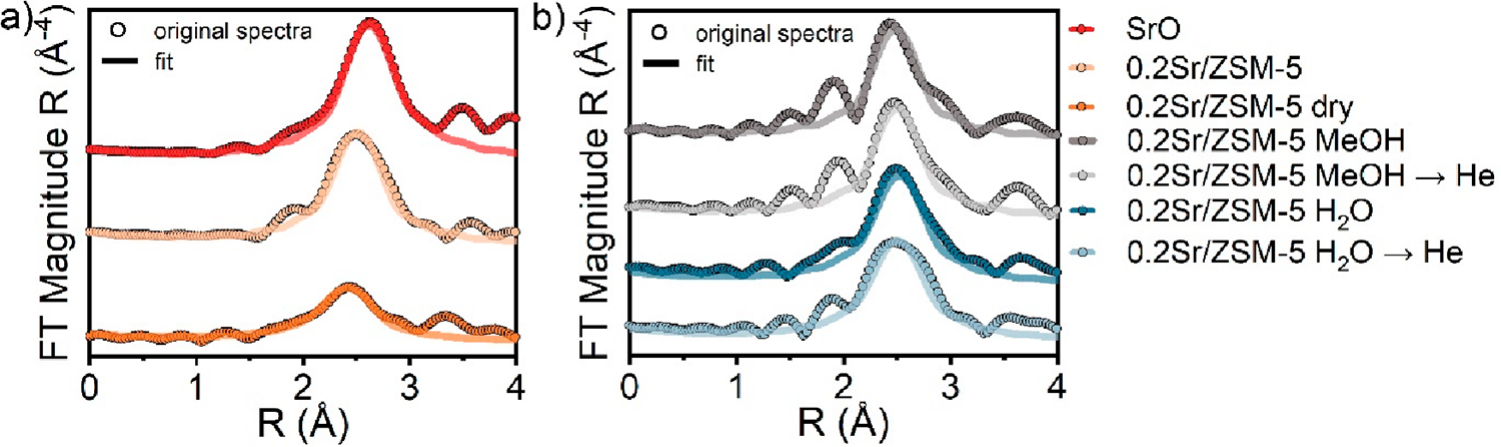
(a, b) Sr K-edge Fourier-transformed EXAFS spectra of SrO and 0.2Sr/ZSM-5 sample with different substrates. Conditions: RT or 50 °C, 25 mg of catalyst, 2.2 kPa of methanol/water, carrier, 30 mL He min^–1^.

Overall, with operando XAS experiments involving switches from a water- or methanol-containing feed to a dry He feed, we established the presence of at least two states of Sr. The EXAFS experiments showed that the Sr coordination number increases in the presence of adsorbates and remains high when the feed is switched to He, indicating that the Sr moieties can strongly retain adsorbates and in this way impact the local environment under the MTH reaction conditions.

We compared the catalytic and structural properties of Sr-modified ZSM-5 catalysts to those of HZSM-5 and Na/ZSM-5 catalysts. STEM imaging of fresh HZSM-5 and used 0.4Sr/ZSM-5 demonstrated that Sr cations occupy a significant fraction of the zeolite pores. Enhanced propylene selectivity and catalyst stability were observed for 0.1Sr/ZSM-5 and 0.2Sr/ZSM-5 catalysts compared to HZSM-5. A further increase in the Sr loading was detrimental for the catalytic activity. Using operando TGA-MS, NMR, IR, and XRD, we established that Sr moieties can affect the reaction via strongly adsorbed water, oxygenates, and possibly hydrocarbons. The amount of retained adsorbates is higher for 0.2Sr/ZSM-5 than for HZSM-5, which is also reflected by a larger and irreversible increase of the unit cell volume of 0.2Sr/ZSM-5 as compared to that of HZSM-5. Following the response to step changes in the reaction feed with XAS showed that the Sr cations can coordinate methanol, water, and hydrocarbons, contributing to the changes in the pore occupancy during reaction. As such, these results corroborate earlier findings for Ca/ZSM-5 that adsorption of reactants and reaction intermediates on alkaline earth metals ions modify the geometry of the zeolite pores in such way that the formation of aromatic hydrocarbon pool species is restricted, resulting in a stronger contribution of the olefinic cycle and a higher selectivity to C_3+_ olefins.

## Supporting Information Available

Catalyst preparation, experimental methods for catalyst characterization and catalytic activity measurements, experimental design schemes, TGA and ^1^H, ^27^Al SS MAS NMR results, operando XRD, IR, XAS results, STEM images, and MCR-ALS analysis (PDF)
