# Supplementary material for: Role of Strontium Cations in ZSM-5 Zeolite in the Methanol-to-Hydrocarbons Reaction
Source: J Phys Chem Lett. 2023 Jul 13;14(28):6506–12. doi: 10.1021/acs.jpclett.3c01259 (PMC10364136; doi:10.1021/acs.jpclett.3c01259)
Supplement: Supplementary file 1 — jz3c01259_si_001.pdf [file jz3c01259_si_001.pdf]

Supporting information

to

On The Role of Strontium Cations in ZSM-5 Zeolite in the Methanol-to-Hydrocarbons Reaction

Anna Liutkova[a], Victor Drozhzhin[a], Jason M.J.J. Heinrichs[a], Valentin Jestl[a], Angelina Evtushkova[a], Brahim Mezari[a], Álvaro Mayoral[b,c], Nikolay Kosinov[a] *, Emiel J.M. Hensen[a] *

[a] Laboratory of Inorganic Materials and Catalysis, Department of Chemical Engineering and Chemistry, Eindhoven University of Technology, P.O. Box 513, 5600 MB Eindhoven, The Netherlands

[b] Instituto de Nanociencia y Materiales de Aragón (INMA), CSIC-Universidad de Zaragoza, 50009 Zaragoza, Spain

[c] Laboratorio de Microscopías Avanzadas (LMA), Universidad de Zaragoza, 50018 Zaragoza, Spain

Corresponding authors:

Nikolay Kosinov

Tel: +31-40-2478156

E-mail: n.a.kosinov@tue.nl

Emiel J.M. Hensen

Tel: +31-40-2475178

E-mail: e.j.m.hensen@tue.nl

Contents

Catalyst preparation S3

Catalyst characterization S3

Catalytic activity measurements S5

Operando measurements: methods S5

X-ray absorption spectroscopy S5

TGA-MS S7

Infrared spectroscopy S7

X-ray diffraction S8

Setup schemes S11

Scanning Transmission Electron Microscopy S12

TGA results S14

Operando IR S15

Operando XRD S16

XAS measurements S18

MCR-ALS analysis of MeOH switching experiment at 450 °C S19

Operando EXAFS S21

References S22

Catalyst preparation

The following zeolite catalysts were prepared for this study: HZSM-5, 0.1Sr/ZSM-5, 0.2Sr/ZSM-5, 0.4Sr/ZSM-5 and Na/ZSM-5. Notations x in Sr/ZSM-5 is the Sr content, i.e., 0.11, 0.22 and 0.44 mmol·g(1). HZSM-5 was obtained by calcining commercial NH_4ZSM-5 zeolite (Si/Al = 25, Alfa Aesar) at 550 °C for 5 h. HZSM-5 modified with Sr were obtained by incipient wetness impregnation of the calcined zeolite with aqueous solutions of $Sr(NO_3)_2$ (Alfa Aesar, 99.0%) following the procedure described elsewhere[1]. The modified catalysts were prepared aiming at Sr loadings of 1, 2, 4 wt.%. The impregnated samples were dried overnight at 110 °C and calcined at 550 °C for 5 h under static air conditions. XRD data demonstrate that the MFI topology of the parent HZSM-5 zeolite is preserved in all samples (Fig. S1). ^{1}H MAS NMR spectroscopy of dehydrated samples was used to compare the amount of Brønsted acid sites (BAS, signal at 4 ppm, Fig. S2). The OH-stretching region of IR spectra of dehydrated samples and IR spectra of adsorbed pyridine provide further information about the acidity situation (Fig. S3). The amount of extraframework Al was estimated using ^{27}Al MAS NMR (Fig. S4). The preparation procedure of ion-exchanged Na/ZSM-5 is provided elsewhere.(2)

Catalyst characterization

The elemental composition (Si/Al ratio and metal content) of the zeolite catalysts was determined by ICP-OES (Spectro CIROS CCD ICP optical emission spectrometer). The samples were dissolved in a 1:1:1 mixture of HF (40%), HNO_3 (65%) and H_2O prior to the ICP measurements.

The crystallinity of the zeolite samples was determined by powder X-ray diffraction (XRD). XRD measurements were performed on a Bruker D2 powder diffraction system (Cu Kα radiation, scan speed 0.01°/s, 2θ range 5−60°).

Solid-state magic angle spinning nuclear magnetic resonance (MAS NMR) spectra were recorded using an 11.7 T AvanceNeo Bruker NMR spectrometer operating at 500 MHz, 125 MHz and 130 MHz for ^{1}H, ^{13}C and ^{27}Al, respectively. ^{1}H and ^{13}C MAS NMR experiments were performed using a Bruker triple channel 4 mm MAS probe head spinning at rates between 8 and 10 kHz. Prior to ^{1}H measurements, the samples were dehydrated and sealed in an air- and moisture-free glovebox. Two-dimensional ^{1}H-^{13}C{1H} HETCOR (HETeronuclear CORrelation) MAS NMR spectra were recorded with a ramped contact pulse time of 5 ms and an interscan delay of 3 s. ^{13}C direct excitation (DE) spectra were measured using a high power proton decoupling Hahn echo pulse sequence p1-τ1-p2-τ2-aq with a 90° pulse p1 = 5 µs, a 180° pulse p2 = 10 µs, a very short τ1= τ2 echo time of 0.013 µs and an interscan delay of 10 s. ^{13}C NMR spectra were recorded at spinning rate of 8 – 10 kHz. ^{27}Al MAS NMR spectra were recorded using a Bruker 2.5-mm MAS probe head spinning at 25 kHz. NMR shift calibration for

^{1}H, ^{27}Al and ^{13}C was done using tetramethylsilane (TMS), saturated $Al(NO_3)_3$ solution, and solid adamantane, respectively.

The acidic properties of zeolites were determined by IR spectroscopy of adsorbed pyridine as a probe molecule. Spectra were taken in the 4000 – 1000 cm^{-1} range using a Bruker Vertex 70v spectrometer. Samples were pressed into self-supporting wafers (10 – 20 mg, diameter 1.3 cm) and placed in an environmental cell. The wafers were pre-treated in O_2:N_2 (1:4 vol. ratio) flow at 550 °C (rate 10 °C·min^{-1}) to remove contaminants followed by cooling to 150 °C under dynamic vacuum ($p < 10^{-5}$ mbar). Afterwards, the samples were exposed to excess pyridine vapor until saturation. IR spectra were then recorded after desorbing pyridine at 150 °C under dynamic vacuum. For the quantification of Brønsted and Lewis acid sites, integral molar extinction coefficients (IMEC) of 0.73 cm·mol^{-1} and 1.11 cm·mol^{-1} were used.(3)

Textural properties of zeolites were determined by Ar porosimetry at -186 °C using a Micromeritics ASAP2020 machine. Prior to measurements, the samples were pre-treated at 400 °C under dynamic vacuum. The micropore volume was calculated by the *t*-plot method using a thickness range from 3.5 to 4.5 Å.

Thermogravimetric analysis (TGA) of used catalysts was performed in a Mettler Toledo TGA/DSC 1 instrument. An amount of used catalyst (≈ 10 mg) was placed in an alumina crucible and heated up to 800 °C at a rate of 5 °C·min^{-1} in a O_2:He (20:40) flow of 60 mL·min^{-1}.

The microscopy experiments were performed using a TITAN X-FEG 60–300 located at the Advanced Microscopy Laboratory, University of Zaragoza, Spain. The microscope is equipped with a field emission gun operating at an acceleration voltage of 300 kV, a CEOS spherical aberration (C_s) corrector for the electron probe, a Gatan Tridiem Energy Filter and an Oxford Silicon drift detector for chemical analysis. Bright and dark field images were simultaneously acquired by annular dark field (ADF) and annular bright field (ABF) detectors. In order to work under very low dose conditions, data acquisition was assisted by the Real Time Up-sampling Filter.(4) The electron dose used for recording the atomic-resolution data was 800–1000 $e^-/Å^2$. For energy-dispersive X-ray spectroscopy (EDX), JEOL EDX spectrometer hardware and JEOL software were employed to acquire and to process the data. The spectra were acquired within 1 min using an energy of 20 keV. Prior to measurements, the samples were crushed, dispersed in ethanol and few drops of the suspension were placed on a holey carbon copper microgrid as described here(5).

Catalytic activity measurements

The catalytic performance in the MTH reaction was performed in a fixed-bed reactor. In a typical experiment, a quartz reactor was charged with 25 mg of the sieved catalyst (250–500 µm pellet size) held between two quartz wool plugs. The catalyst was subsequently pre-treated in an oxygen atmosphere (20 vol.% O_2 in He) at 550 °C (ramp rate 10 °C·min^{-1}) for 1 h to remove organic contaminants. After pre-treatment, the temperature was set to 450 °C in pure He. The reaction was started by changing the feed to a 30 mL·min^{-1} flow of methanol in He (the temperature of the thermostat was kept at 19 °C, resulting in a methanol partial pressure of 12 kPa) at a WHSV 12 h^{-1}. The reactor outlet was connected to a GC (Compact GC 4.0, Global Analyzer Solutions) and an MS (Pfeiffer Omnistar) with heated gas transfer lines. The GC was equipped with two pre-columns, three columns and three detectors. A thermal conductivity detector (TCD) coupled with an RT-Q-Bond pre-column (length 3 m; i.d. 0.32 mm; film thickness 10 µm) and a Molsieve 5A FS column (Restek, length 10 m; i.d. 0.32 mm; film thickness 30 µm) was used for the analysis of the light reaction products (H_2, CH_4). Light hydrocarbons (C_2 – C_3), water, and oxygenates were analyzed by another TCD coupled with a RT-Q-Bond pre-column (length 3 m; i.d. 0.32 mm; film thickness 10 µm) and an RT-Q-Bond column (Restek, length 10 m; i.d. 0.32 mm; film thickness 10 µm). Heavier hydrocarbons (C_4 to trimethylbenzenes) were separated using an Rtx-1 column (Restek, length 15 m; i.d. 0.32 mm; film thickness 1 µm) and analyzed with a flame ionization detector (FID). Conversion was defined as the carbon-based fraction of oxygenates (methanol and dimethyl ether) consumed during the reaction. Selectivity to products was calculated on the carbon atom basis. Methanol throughput is defined as the mass of methanol converted per mass of catalyst before the conversion of methanol and DME decreased below 75%). Overall carbon selectivity during the MTH reaction was provided after 1 h time on stream. Conversion of methanol was calculated using the following formula:

$$X = \frac{n_{C,MeOH_{in}} - n_{C,MeOH_{out}} - 2 \times n_{C,DME_{out}}}{n_{C,MeOH_{in}}} \times 100\% \qquad (1.1)$$

Further calculation procedure was reproduced from here.(1,6)

Operando measurements: methods

X-ray absorption spectroscopy

Operando X-ray absorption spectroscopy (XAS) experiments were performed at the Sr K-edge (16105 eV) in transmission mode at the P65 beamline of the Petra III synchrotron in Hamburg (Germany) and at the BL22 (CLÆSS) beamline of the ALBA synchrotron in Barcelona (Spain). For the in situ

measurements at P65, a double crystal Si (311) monochromator was used and the beam size was 0.2 mm × 1.0 mm.(7) An Y foil (Y K-edge, 17037 eV) was measured in transmission mode for energy calibration of the data. Complementary ex situ measurements at BL22 also made use of a double crystal Si (311) monochromator with a beam size of 225 × 56 μm^2.(8)

For the operando measurements at P65, dynamic switches of the feed (methanol/He → He, water/He → He) at 4 temperatures (i.e., 50, 200, 350 and 450 °C) were combined with XAS and mass spectrometry (MS) measurements. A modified oven with a ~1 cm × 1 cm aperture for transmission of X-rays was used (Fig. S5b). In a typical experiment, 25 mg of catalyst in a sieved fraction of 250 – 500 µm were placed in a quartz tube (length 250 mm, i.d. 4.0 mm, o.d. 5.0 mm), with a flattened middle section (length 20 mm, thickness 0.25 mm, Fig. S5c).(9) Prior to every measurement, the catalyst was treated at 500 °C for 30 min in He:O_2 flow (1:3). Then the catalyst was cooled to the measurement temperature and blank spectra of the dried zeolite were recorded. We used an automated 4-way valve to switch the feed every 20 min from dry He to a substrate-containing He flow. The scheme of the data collection is provided in Fig. S5a. In a typical step-response experiment, a thermostated saturator was used to supply methanol or water vapors to the catalyst bed: a He flow of 30 $mL \cdot min^{-1}$ was led through the saturator in the case of both methanol and water experiments. For the methanol switches the temperature of the thermostat was kept at -9.1 °C, resulting in a methanol partial pressure of 2.2 kPa. For the water switches, the temperature of the thermostat was kept at 19 °C, resulting in a water partial pressure of 2.2 kPa. Calibrated thermal mass-flow controllers (Brooks) were used to supply the gases to the reactor oven. The outlet of the reactor was connected to an MS (Pfeiffer Omnistar MS). A scheme of the setup and photographs of the experimental stage and the reactor are provided in Figs. S5b-c, S6.

SrO, $Sr(OH)_2$, $Sr(OH)_2 \cdot 8H_2O$, $C_4H_6O_4Sr$, $SrCO_3$ and $Sr(NO_3)_2$ were used as reference compounds (XANES spectra provided in Fig. S17). Prior recording ex situ XAS spectra, these compounds were diluted with an appropriate amount of boron nitride and pelletized. Data alignment and merging were performed with the Athena software.(10) The contribution of different Sr-containing phases was resolved using multivariate curve resolution with alternating least squares (MCR-ALS) analysis of XANES data using spectra of the dried zeolite and the zeolite in hydrated form, obtained from an experiment upon switching to a water-containing feed at 450 °C. MCR-ALS analysis was performed with the MAX (Multiplatform Application for XAFS) software package.(11) The procedure of the analysis was reproduced from literature.(12) Non-negative constraints were applied to pure component spectra and their fractions, while the cumulative contribution profile closure was set to unity. As a convergence criterion for MCR-ALS analysis, a standard deviation of the residuals lower than or equal to 0.1% between two consecutive iterations was used. Reported EXAFS spectra for the SrO reference

and 0.2Sr/ZSM-5 are the average of two measurements (Fig. S5a). Background subtraction and Fourier-transformation was done in Athena, while fitting was done in Artemis, both of which are interfaced to the IFEFFIT package (version 1.2.12).(10) Scattering Sr-O paths were calculated in Artemis using the crystal structure of SrO obtained from its CIF file (Material Project, mp-2472) (13). The coordination number (N), change in bond distance (ΔR), displacement (σ^2), and energy shift (ΔE_0) were fitted. The fitting ranges were $\Delta k = 3 – 12$ $Å^{-1}$ and $\Delta R = 1.3 – 2.6$ Å. The spectra presented are k^3-weighted and phase-corrected. Amplitude reduction factors were extracted from fitting of the EXAFS spectrum of SrO.

TGA-MS

The procedure for TGA-MS analysis is analogous to the one we have previously reported[2]. In short, we used a Mettler Toledo TGA/DSC 1 instrument connected to an MS (Pfeiffer Omnistar). A typical amount of 10 mg of catalyst was placed in an alumina crucible and pre-treated in O_2:He (1:3 vol. ratio) flow at 550 °C (rate 10 °C·min^{-1}) for 1 h to remove organic contaminants, followed by cooling to the reaction temperature of 350 °C and start of the TG analysis. In a typical experiment, an automated 4-way valve was used to switch the feed every 20 min from dry He to a MeOH-containing He flow. A thermostated saturator was used to supply methanol vapor to the catalyst bed. For this, a He flow of 40 mL·min^{-1} was led through a saturator followed by dilution with another He flow of 40 mL·min^{-1}. The temperature of the thermostat was kept at -14.6 °C, resulting in a methanol partial pressure of 1.5 kPa. After dilution with the additional He flow, the methanol partial pressure was 0.75 kPa. A dry He flow of 80 mL·min^{-1} was supplied to the catalyst bed for dry conditions. Calibrated thermal mass-flow controllers (Brooks) were used to supply the gasses to the TGA chamber.

Infrared spectroscopy

To study the response of the catalysts to the switches between methanol and dry He by IR spectroscopy, we used a Bruker Vertex 70v IR spectrometer to which a setup was connected for methanol dosing.(2) Spectra were taken in the 4000 – 1000 cm^{-1} range. Samples were pressed into self-supporting wafers (10 – 20 mg, diameter 1.3 cm) and placed in an environmental cell. The wafers were pretreated in O_2:He (1:2 vol. ratio) flow at 550 °C (rate 10 °C·min^{-1}) to remove organic contaminants, followed by cooling to 350 °C in He. After that, IR measurements were started. First, background spectra were recorded. An automated 4-way valve was used to switch the feed every 20 min from dry He to a MeOH-containing He flow. For this purpose, a thermostated saturator was used to supply methanol vapor to the catalyst bed: a He flow of 10 mL·min^{-1} was led through the saturator followed by dilution with another He flow of 120 mL·min^{-1}. The temperature of the thermostat was

kept at −14.6 °C, which yielded after dilution with the additional He flow in a methanol partial pressure of 0.12 kPa. A dry He flow was fed at a rate of 130 mL·min^{-1} to obtain dry conditions. Calibrated thermal mass-flow controllers (Brooks) were used to supply the gases to the IR cell. The outlet of the IR cell was connected to an MS (Pfeiffer Omnistar MS).

X-ray diffraction

Operando XRD experiments were performed at the ID31 beamline of ESRF synchrotron (Grenoble, France).(2) The photon wavelength was 0.0165 nm (75 keV) with an unfocused beam of 0.5 mm × 0.5 mm, and a Pilatus3 X CdTe 2M X-ray detector (Dectris) was used. Sieved (250 – 500 µm) ZSM-5 catalyst (20 mg) was placed in a quartz capillary (i.d. 2.8 mm, o.d. 3.0 mm, wall thickness 0.1 mm) to form a catalyst bed of 4 mm in length. The capillary was sealed by PTFE ferrules in a home-built Clausen-type flow cell,(14) located on a movable sample stage. The catalyst bed was heated to 550 °C using two gas blowers (Cyberstar) for the pre-treatment in artificial air flow 50 ml·min^{-1} for 1 h and then cooled to the reaction temperature of 400 °C. The temperature was controlled by a thin (0.25 mm) K-type thermocouple placed inside the catalyst bed. After the temperature reached 400 °C and was stabilized for 30 min, blank diffractograms were recorded and 50 ml·min^{-1} He flow along with evaporated methanol and Ar tracer 2 ml·min^{-1} was supplied to the catalyst bed. The temperature of the thermostat was kept at 20 °C, resulting in a methanol partial pressure of 13 kPa. The chemical composition of the outlet flow was analyzed by a quadrupole mass-spectrometer (QGA, Hidden Analytica). The capillary was moved up and down during the experiment to acquire diffractograms at different positions of the bed. The integrated XRD patterns were analyzed by Rietveld refinement using the GSAS-II software. The patterns were refined in q-range of 0.4 – 6 Å^{-1}. The scale factor, background, and the unit cell parameters (P*nma* space group) were refined. Other parameters (crystal size, strain, displacement, atomic and thermal parameters, etc.) were initially refined for the activated HZSM-5 sample and kept the same for each pattern during the following refinement.

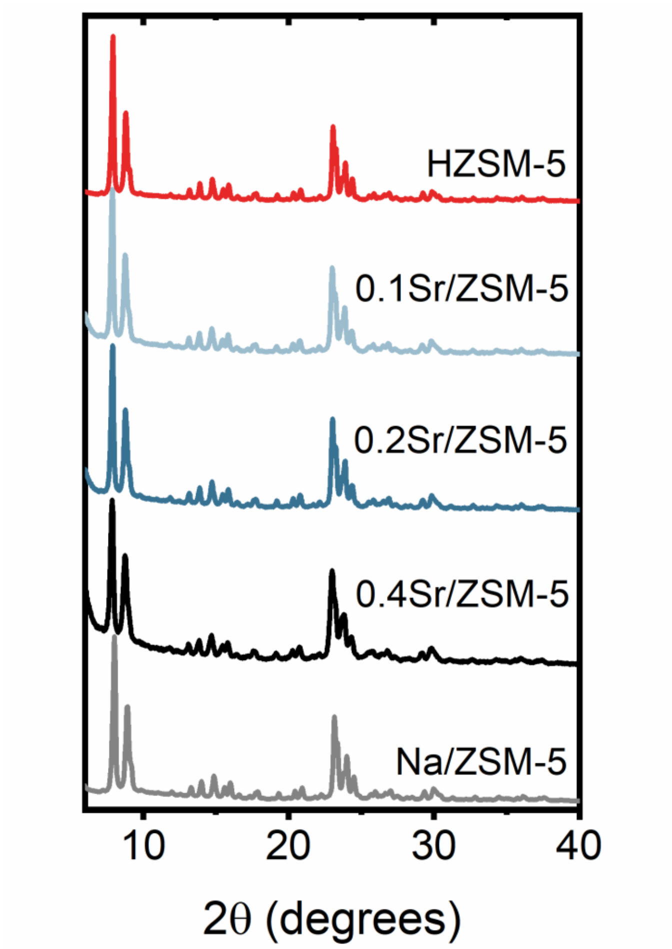

Figure S1. XRD patterns of the prepared catalysts (Cu Kα source).

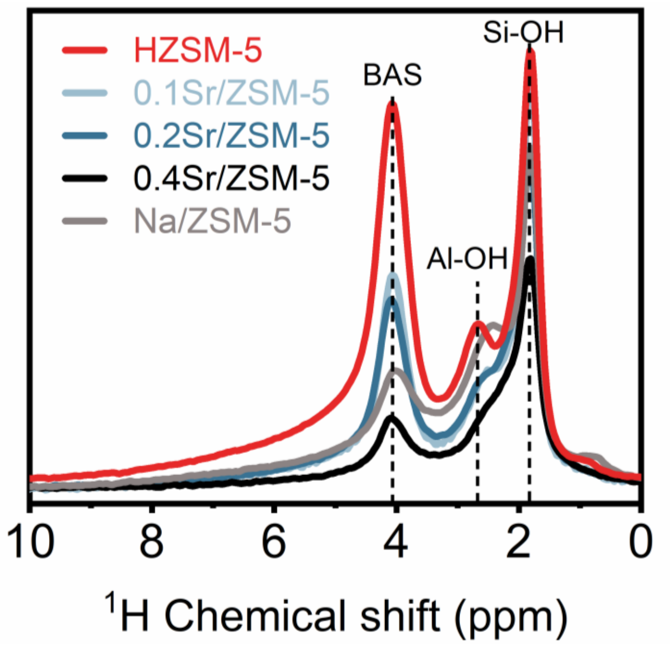

Figure S2. ^{1}H MAS NMR spectra of the calcined catalysts. The peaks with a chemical shift of 4 and ~6 ppm correspond to isolated and H-bonded bridged Si-O(H)-Al. The signals at 2.6 ppm and 1.8 ppm can be assigned to extra-framework Al-OH and Si-OH groups, respectively.

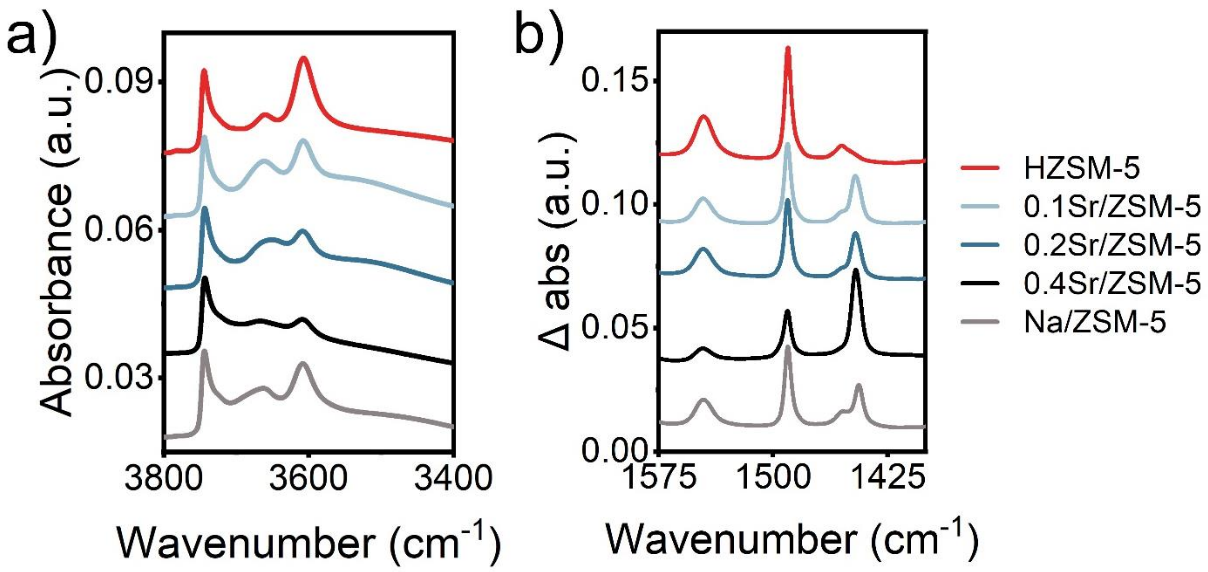

Figure S3. OH-stretching region before pyridine adsorption (a) and pyridine ring vibration region (b) after pyridine adsorption and desorption at 150 °C.

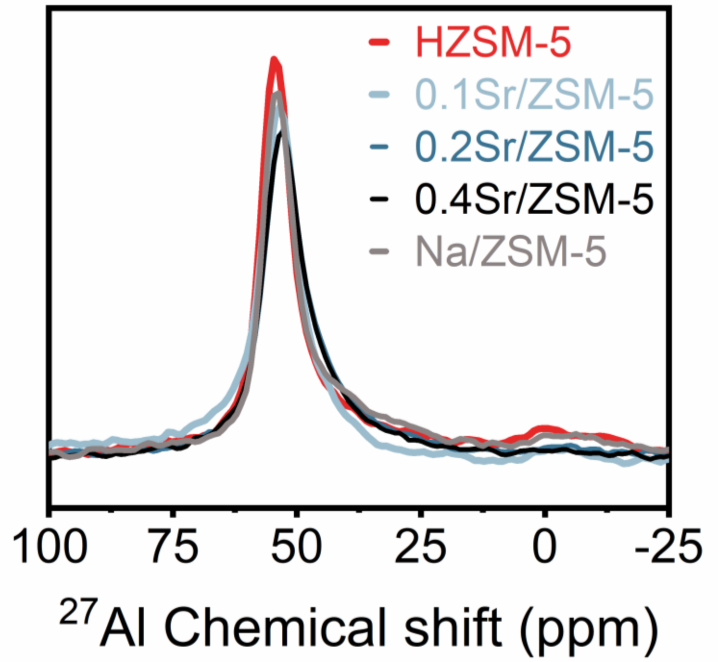

Figure S4. ^{27}Al MAS NMR spectra of the prepared catalysts. The peak at 53 ppm and 0 ppm correspond to tetrahedral framework Al and octahedral extra-framework Al, respectively.

Setup schemes

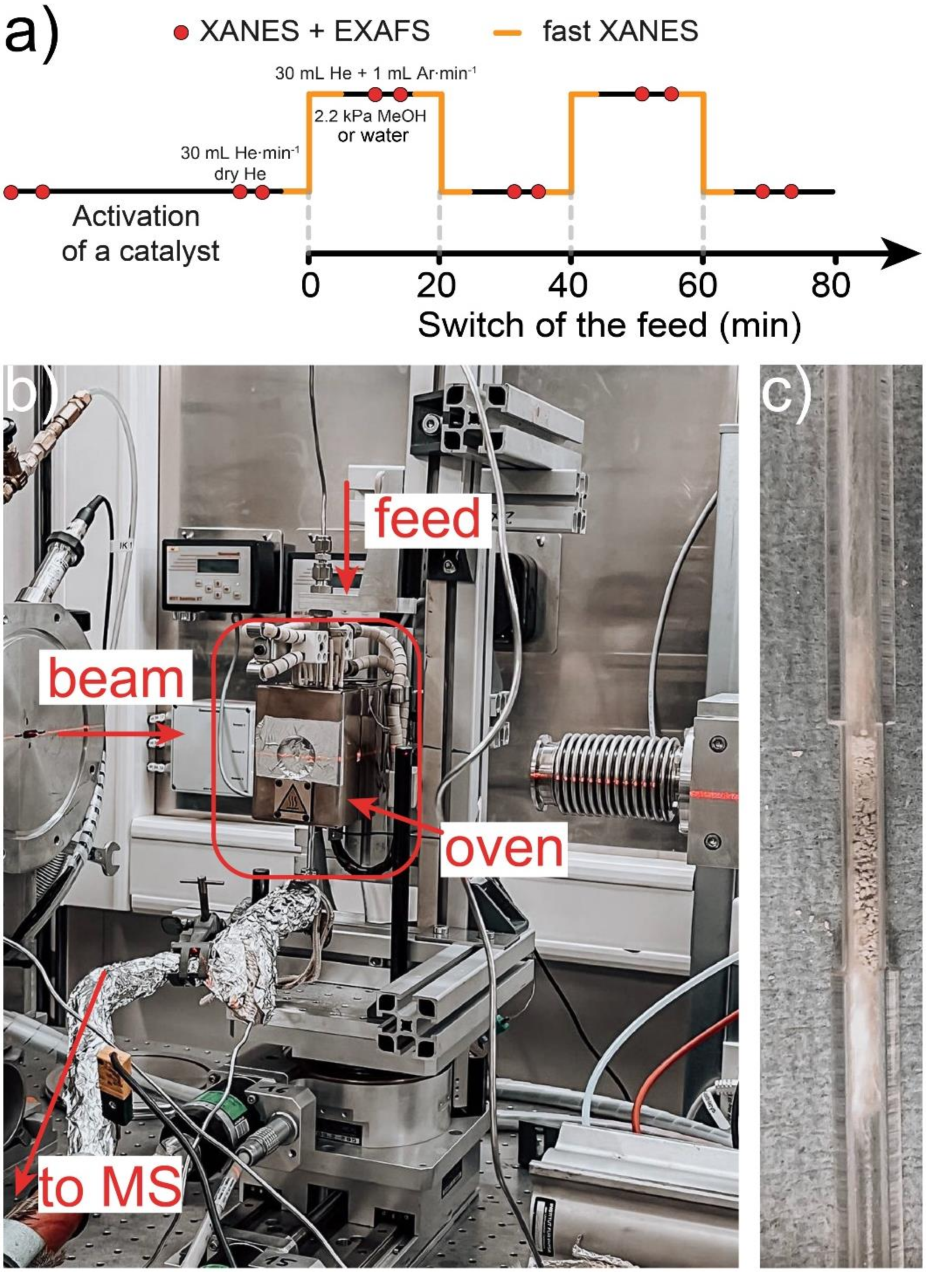

Figure S5. (a) Design of the XAS experiment; (b) experimental setup at beamline P65, Petra III and (c) detail of flattened quartz reactor for XAS switches.

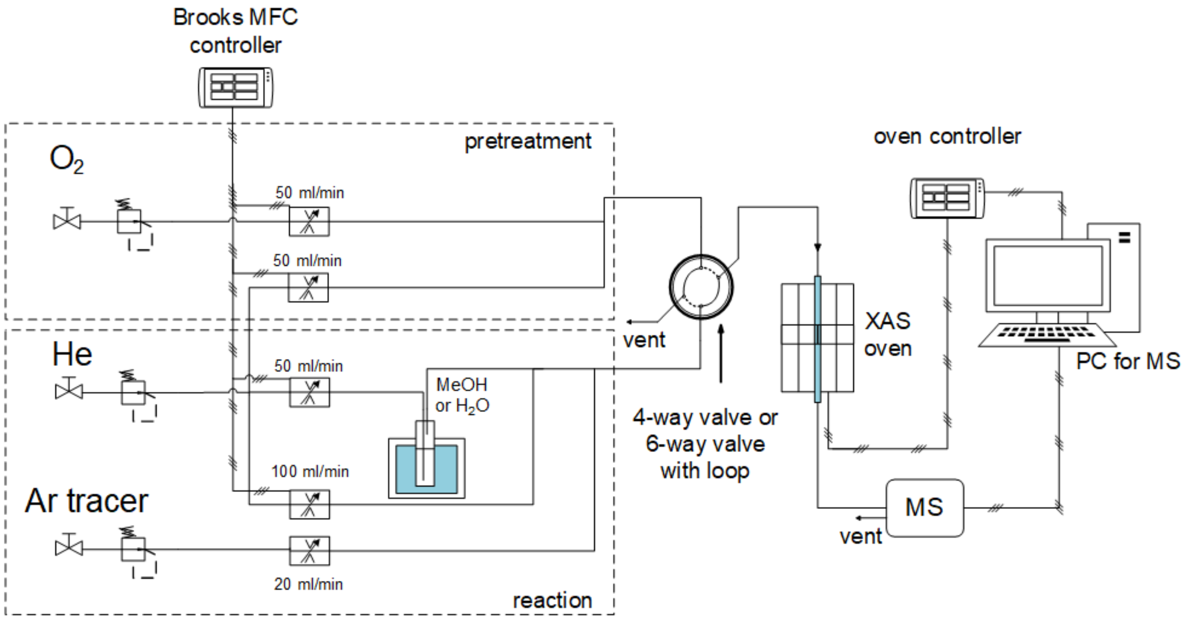

Figure S6. Scheme of the setup used for step-response XAS experiments.

Scanning Transmission Electron Microscopy

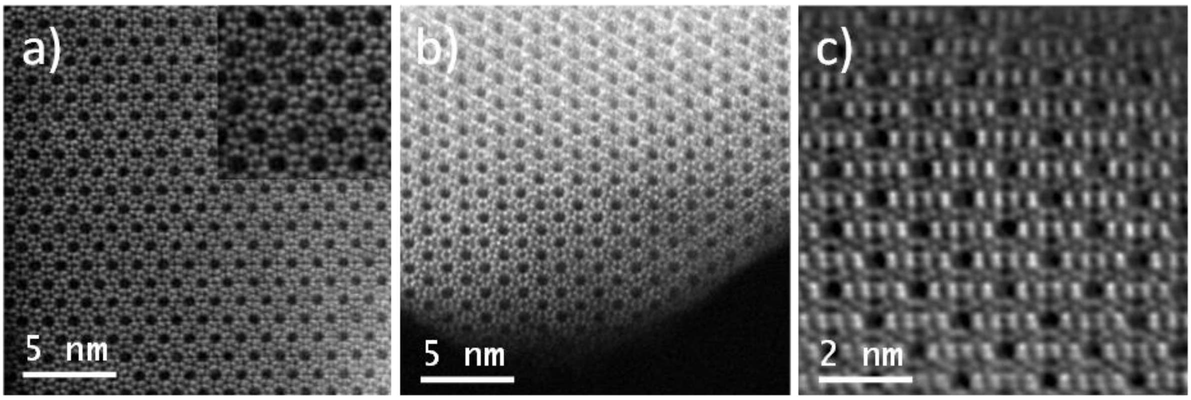

Figure S7. C_s-corrected STEM micrographs of fresh HZSM-5 zeolite along the [010] zone axis. (a) High-resolution STEM-ADF image of the framework with an enlarged view shown in the inset. (b) High-resolution STEM-ADF image of another crystal. (c) Contrast inverted STEM-ABF image along the [100] zone axis of another crystal.

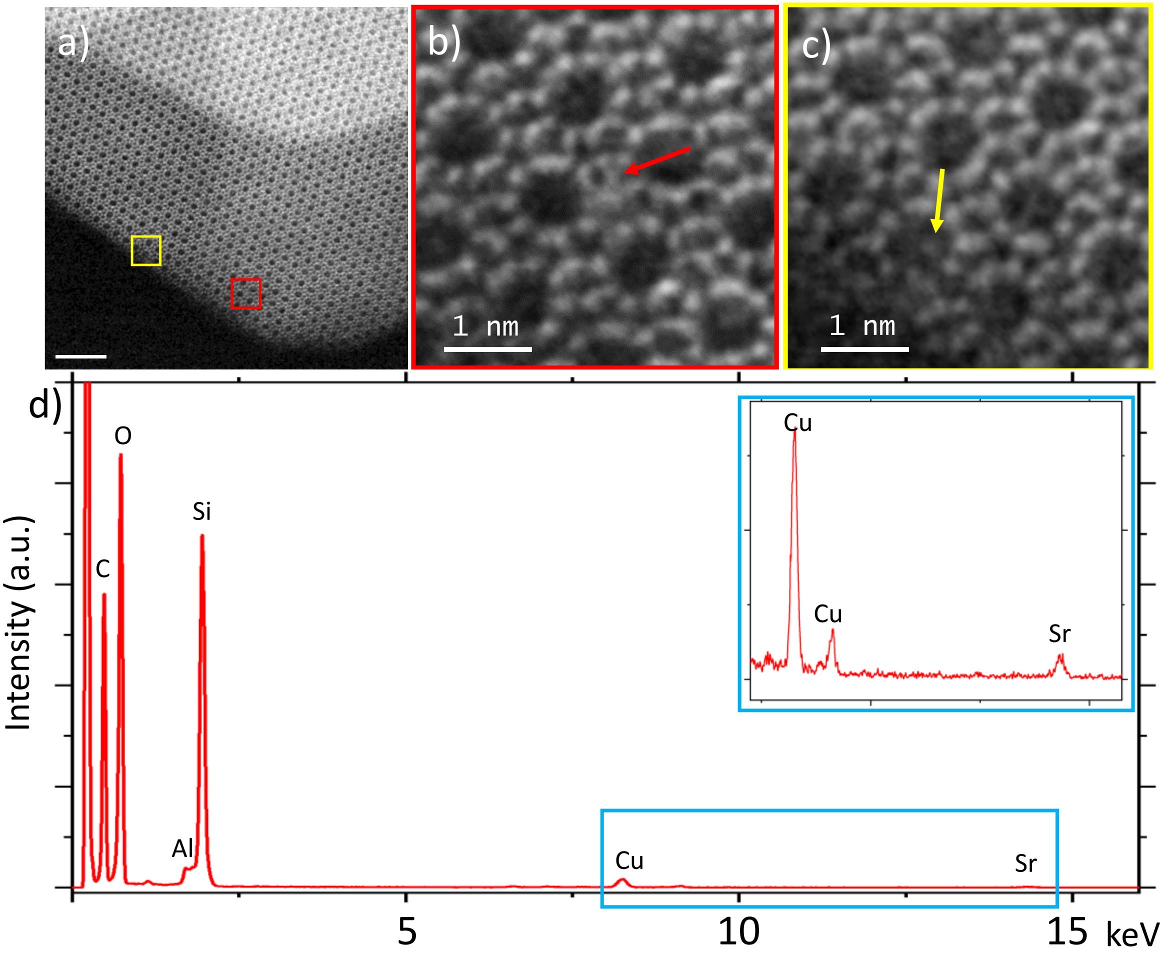

Figure S8. C_s-corrected STEM-ADF micrographs along the [010] zone axis of used 0.4Sr/ZSM-5. (a) High-resolution image which allows the framework visualization, with the two regions magnified indicated by red and yellow squares. (b and c) Magnified views, at atomic-resolution level, of the two regions marked in (a) where the extraframework Sr species are indicated by red (in 6 membered rings) and yellow (10-membered rings), respectively. (d) EDS spectrum profile showing the existence of Sr signal. C and Cu signals correspond to the grids used. The used 0.4Sr/ZSM-5 was obtained after reaction for 1 h on methanol stream, 450 °C, 25 mg of catalyst, 12 kPa of MeOH, carrier – 30 mL·min^{-1} He, WHSV 12 h^{-1}.

TGA results

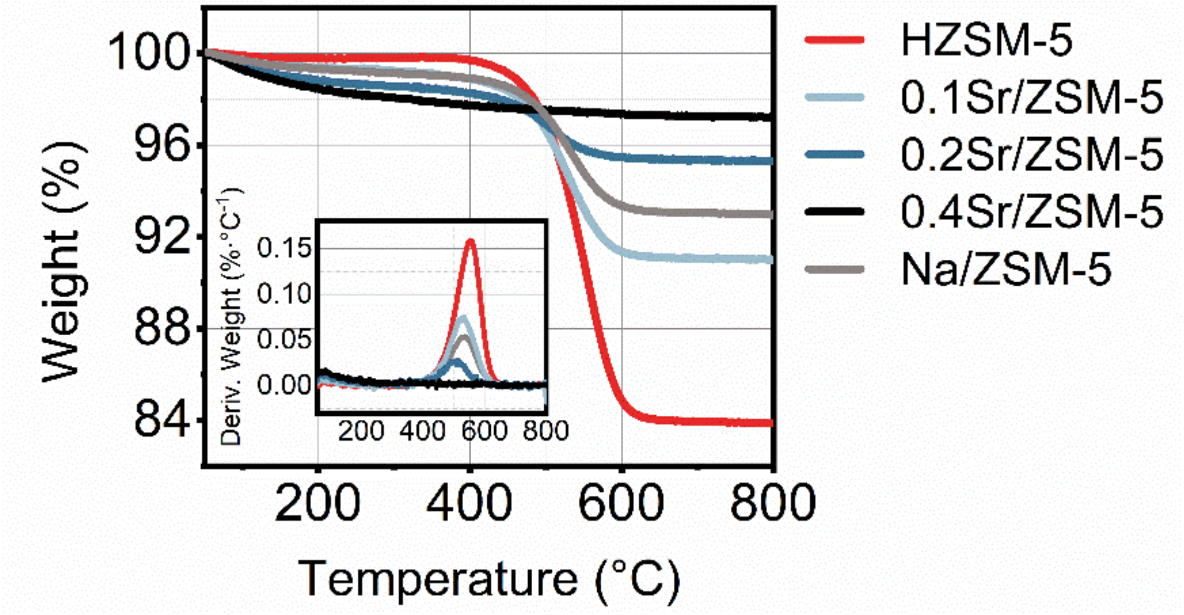

Figure S9. TG and DTG (inset) analysis of spent catalysts. Deactivated samples were obtained at the following reaction conditions: 25 mg of catalyst, 12 kPa of MeOH, carrier – 30 mL·min^{-1} He, WHSV 12 h^{-1}.

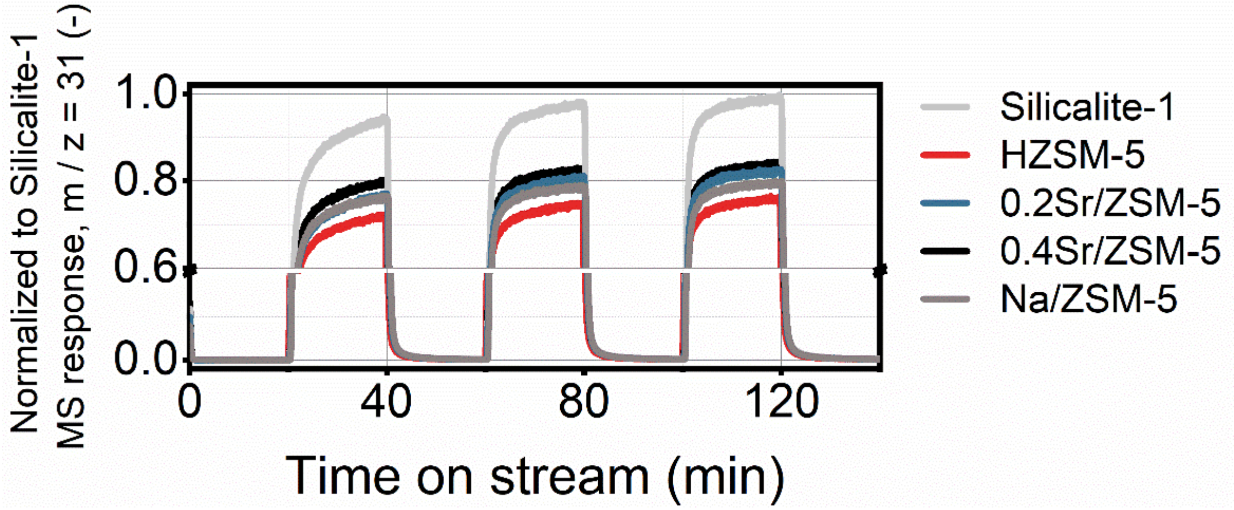

Figure S10. MS profiles of *m/z* = 31 signal corresponding to methanol recorded simultaneously with TG profiles in Fig. 1c. Conditions: 350 °C, 10 mg of catalyst, carrier – 80 mL·min^{-1} He, 0.75 kPa of MeOH. Properties of Silicalite-1 material and its synthesis procedure can be found in the literature.(15)

Operando IR

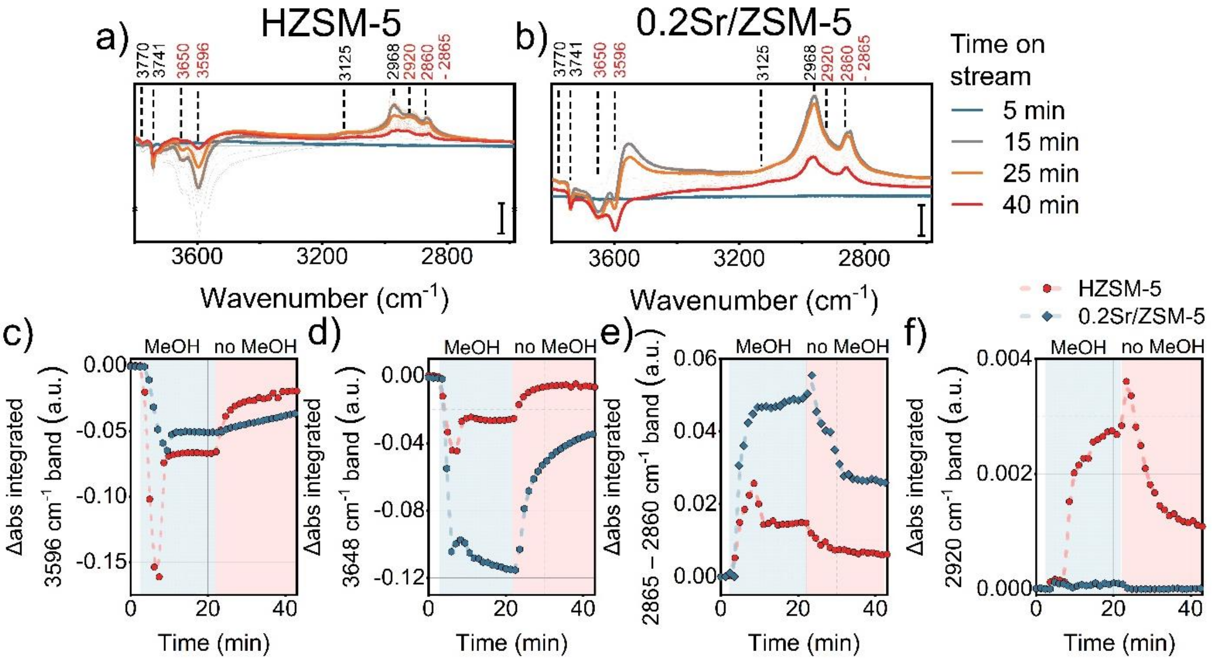

Figure S11. (a,b) Operando IR measurements over ZSM-5 catalysts in presence (5 min and 15 min on stream) and absence (25 min and 40 min on stream) of the methanol feed and corresponding Δ abs spectra: OH-region and methanol adsorption region. The Δ abs spectra were obtained by subtraction of the first spectrum recorded at 350 °C in absence of methanol from all other spectra. Conditions: 350 °C, 15 mg of catalyst pellet, carrier – 130 mL·min^{-1} He, 0.12 kPa of MeOH. (c-f) Bands of interest integrated from Δ abs spectra from operando IR measurements for ZSM-5 catalysts in presence and absence of the methanol feed: (c) BAS, (d) Al-OH and Sr-OH, (e) methoxy groups, (f) polymethylated benzenes.

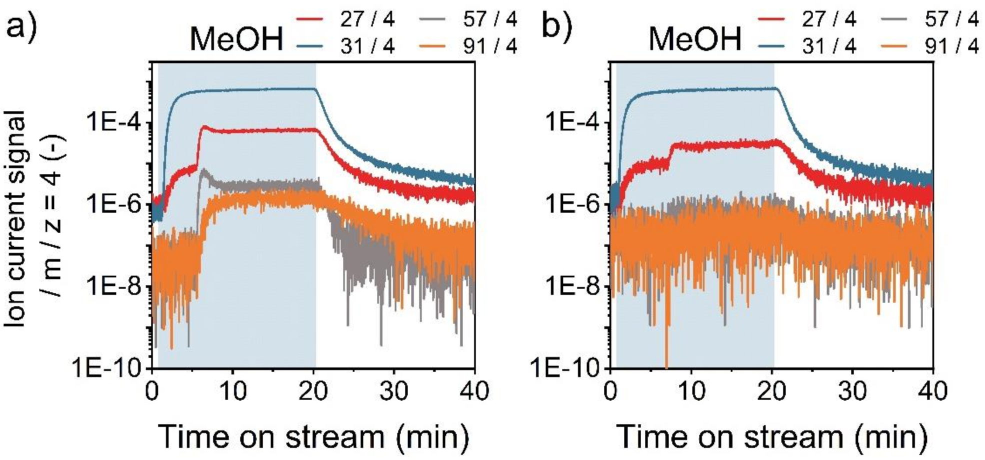

Figure S12. MS profiles for IR experiments normalized per m/z = 4 signal (He), signal m/z = 27 corresponds to ethylene, m/z = 31 – methanol, m/z = 57 – butane, and m/z = 91 to toluene. Conditions: 350 °C, 15 mg of catalyst pellet, carrier – 130 mL·min^{-1} He, 0.12 kPa of MeOH.

Operando XRD

We performed operando XRD analysis over HZSM-5 and 0.2Sr/ZSM-5. The changes in the zeolite unit cell volume were determined by Rietveld refinement of the experimental patterns. We found that the unit cell of Sr-modified catalyst expands more upon filling with adsorbates during 5 h on methanol stream as compared to HZSM-5 (Fig. S13). After the methanol was switched off, the unit cell of Sr-free HZSM-5 became smaller again, while the unit cell of 0.2Sr/ZSM-5 remained nearly unchanged, indicating that the adsorbates were retained in an irreversible manner over Sr-modified zeolite. By following 5 different positions along the catalyst bed, we established that the expansion of the unit cell volume at the beginning of the catalyst bed is faster, which suggests a faster build-up of hydrocarbon pool species in the active zone of the catalyst close to the reactor inlet.

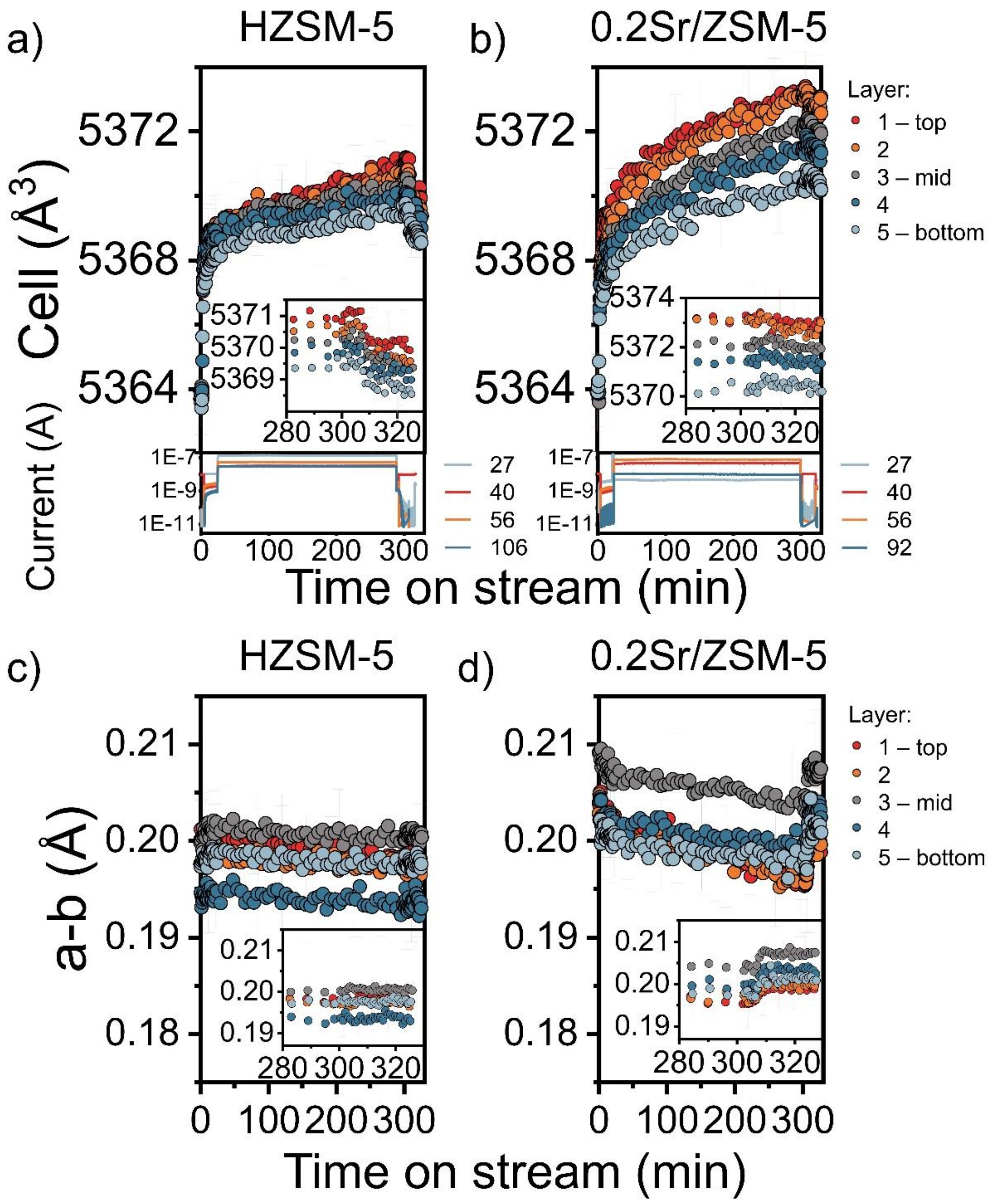

Figure S13. (a – b) Unit cell volumes derived from Rietveld refinement of operando XRD data for HZSM-5 and 0.2Sr/ZSM-5 catalysts and after 5 h on stream and subsequent switch off the methanol for 30 min; MS spectra of the reaction are attached below, m/z = 27 corresponds to ethylene, m/z = 40 – Ar, m/z = 56 – butene, 92 – toluene. (c – d) Difference of unit cell vectors a and b derived from Rietveld refinement. Conditions: 400 °C, 20 mg of catalyst, 13 kPa of MeOH, carrier – 50 mL^{-1}·min He.

XAS measurements

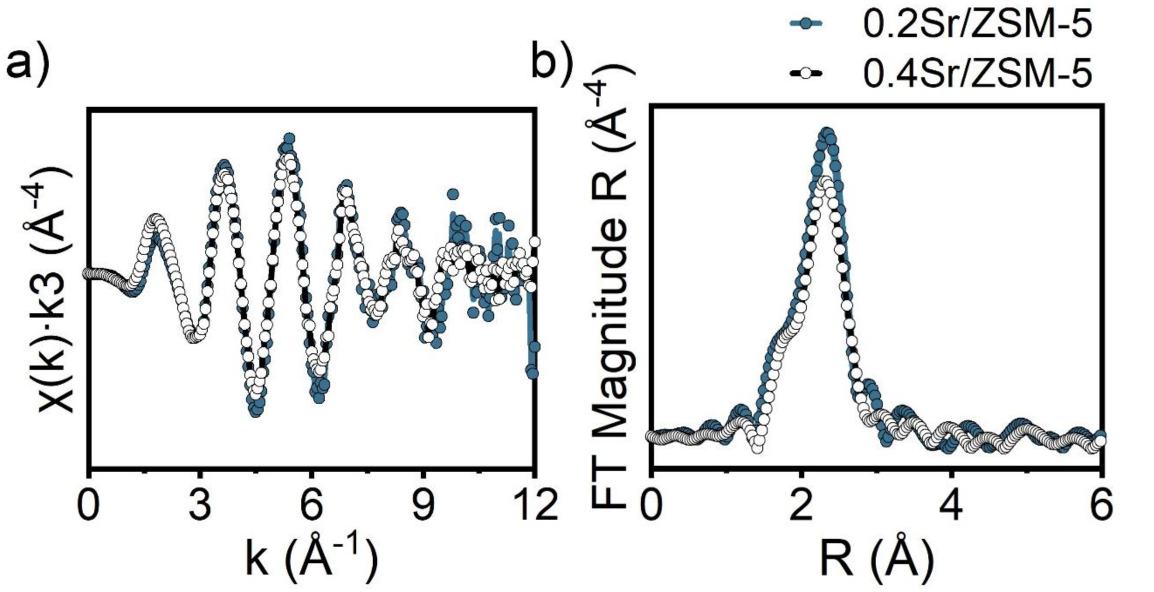

Figure S14. EXAFS analysis of Sr K-edge of 0.2Sr/ZSM-5 and 0.4Sr/ZSM-5 samples: (a) EXAFS χ(k) plots, (b) Fourier-transformed EXAFS spectra.

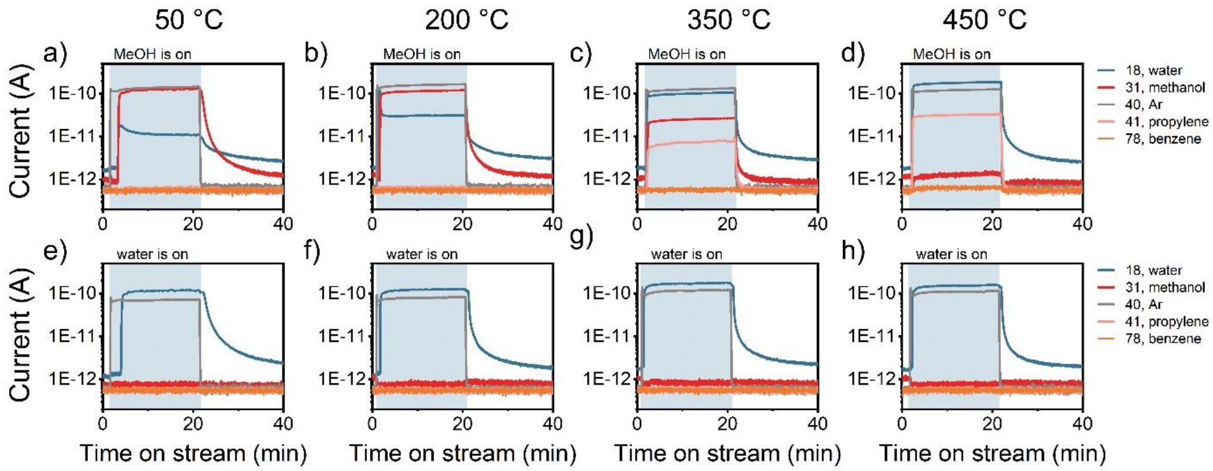

Figure S15. MS response over 50, 200, 350 and 450 °C XAS switching experiments: (a-d) methanol switches, (e-h) water switches.

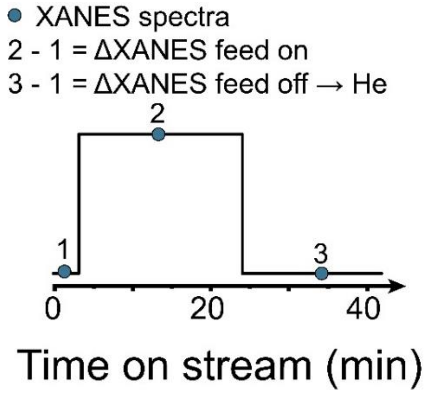

Figure S16. XANES data collection principle used for the comparison of ΔXANES spectra after different catalyst pretreatments.

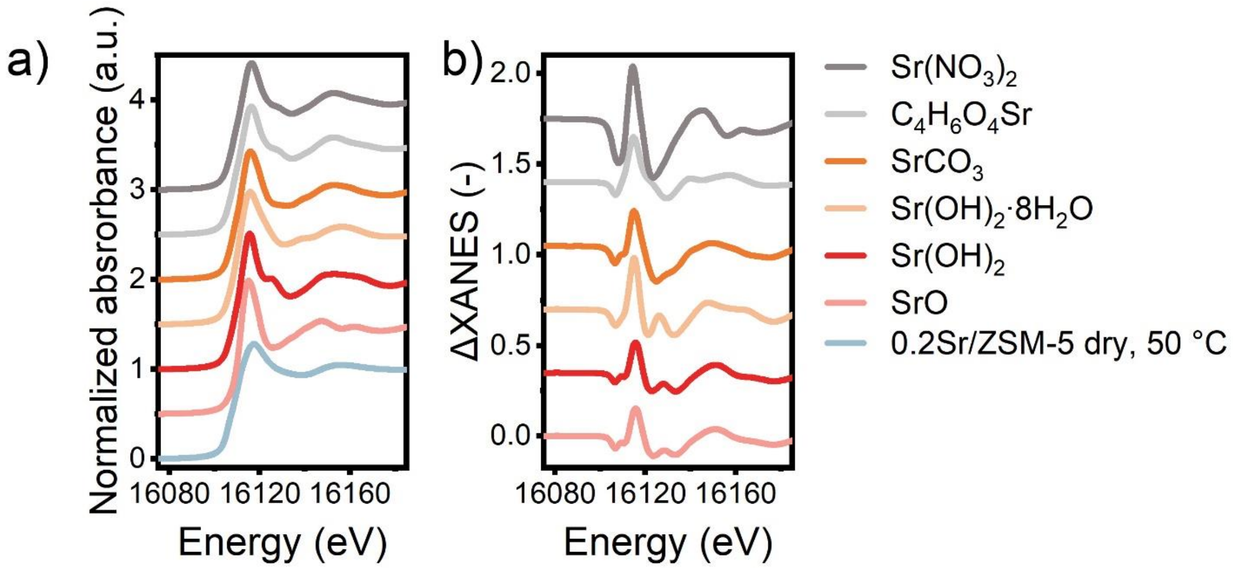

Figure S17. (a) XANES spectra of references measured at room temperature and activated (dry) 0.2Sr/ZSM-5 cooled down to 50 °C; (b) its corresponding ΔXANES obtained by subtraction of the activated 0.2Sr/ZSM-5.

MCR-ALS analysis of MeOH switching experiment at 450 °C

The detailed procedure of the analysis is ascribed here.(12,16) In short, using MCR-ALS we were able to distinguish the presence of two states of Sr upon MeOH/He → He switch at 450 °C (Figs. S18 – S19). We assigned them to “dry” Sr and hydrated Sr, using activated 0.2Sr/ZSM-5 and watered 0.2Sr/ZSM-5 and later on, SrO and $Sr(OH)_2 \cdot 8H_2O$ (Fig. 3c) as references.

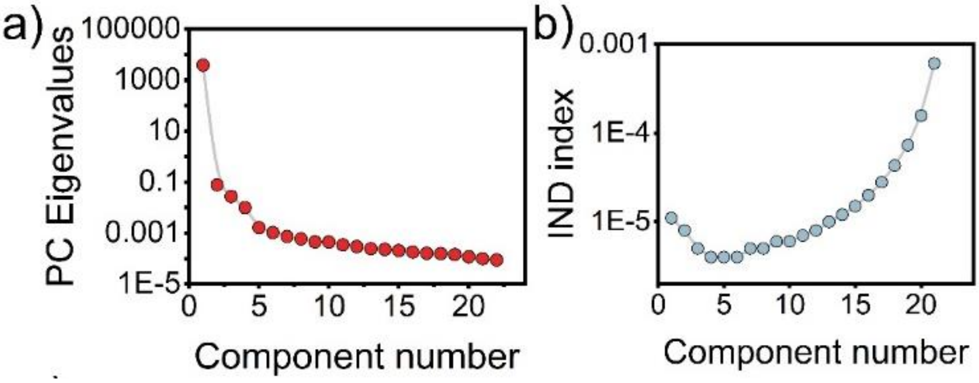

Figure S18. (a) Scree plot of PC eigenvalues obtained from PCA analysis and (b) IND factor.

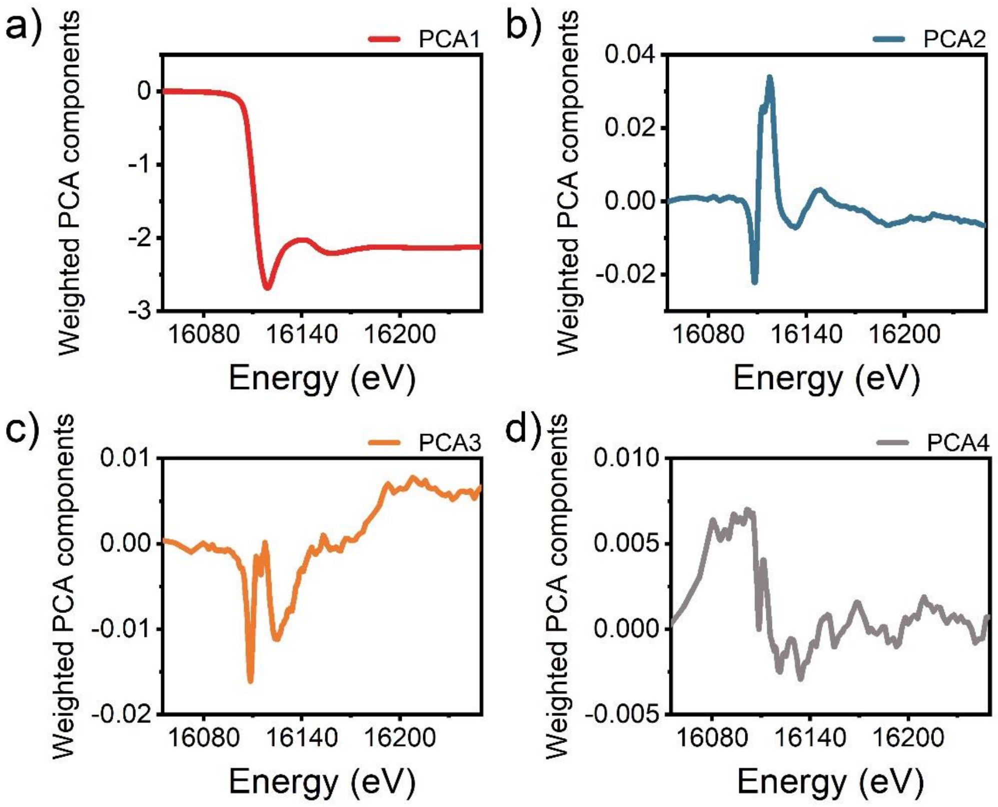

Figure S19. Plot of the first four PCA components weighted by their respective eigenvalues for 450 °C MeOH switch experiment. The eigenvalue of each component is 3860.403 for PCA1, 0.078 for PCA2, 0.027 for PCA3 and 0.010 for PCA4.

Operando EXAFS

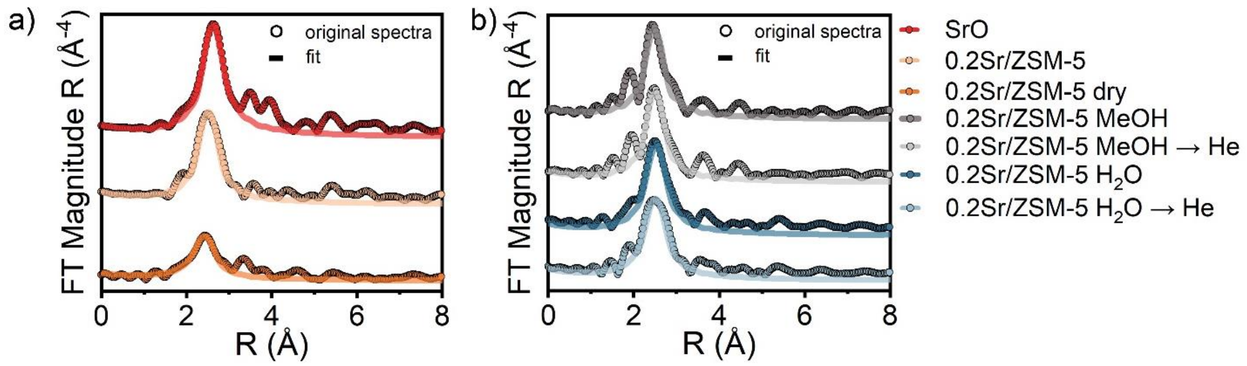

Figure S20. Sr K-edge Fourier-transformed EXAFS spectra of SrO and 0.2Sr/ZSM-5 sample at different substrates, wider range. Conditions: RT or 50 °C, 25 mg of catalyst, 2.2 kPa of methanol or water, carrier – 30 mL He·min^{-1}.

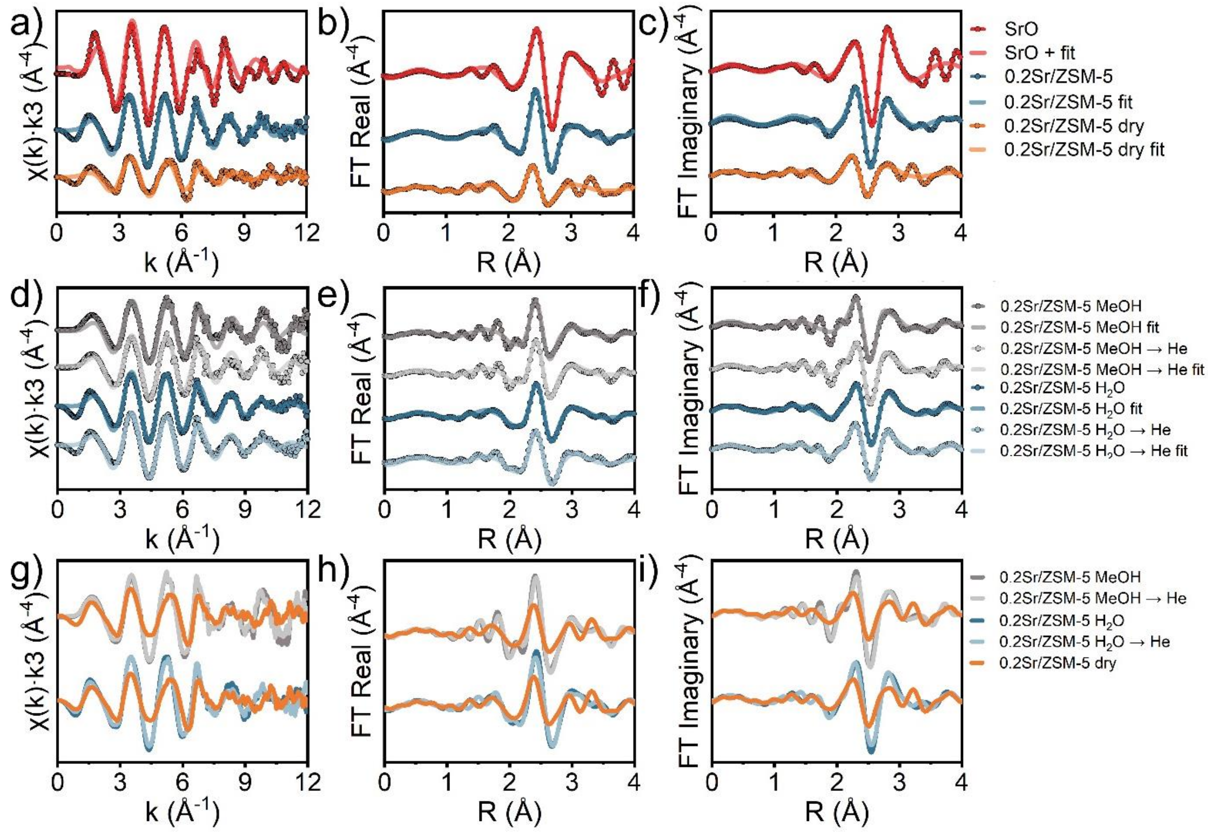

Figure S21. EXAFS analysis of SrO and 0.2Sr/ZSM-5 samples at different conditions. (a,d,g) EXAFS χ(k) plots, (b,e,h) real parts of the Fourier transform, (c,f,i) imaginary parts of the Fourier transform.

Table S1. EXAFS fit with uncertainties for SrO and 0.2Sr/ZSM-5.

Sample	Substrate	T (°C)	$N1_{Sr-O}$ (-)	$R1_{Sr-O}$ (Å)	σ^2_{Sr-O} (Å^2) $\cdot 10^{-2}$	E_0 (eV)	R factor (%)
**SrO**	Air	25	**8.0**	2.6 ± 0.1	1.2	2.3	0.3
**0.2Sr/ZSM-5**	Air	25	**6.4 ± 0.5**	2.5 ± 0.2	1.2	-4.8	0.6
	Dehydrated (dry), He	50	**3.9 ± 0.6**	2.5 ± 0.3	1.4	-8.2	1.5
	Water	50	**6.5 ± 0.4**	2.5 ± 0.2	1.2	-3.4	0.3
	Switch to He from water, He	50	**6.4 ± 0.6**	2.5 ± 0.2	1.3	-4.1	2.5
	Methanol	50	**5.4 ± 0.9**	2.5 ± 0.2	1.0	-4.6	4.3
	Switch to He from methanol, He	50	**5.5 ± 0.8**	2.5 ± 0.2	1.0	-4.0	3.0

References

(1) Yarulina, I.; Bailleul, S.; Pustovarenko, A.; Martinez, J. R.; Wispelaere, K. De; Hajek, J.; Weckhuysen, B. M.; Houben, K.; Baldus, M.; Van Speybroeck, V.; Kapteijn, F.; Gascon, J. Suppression of the Aromatic Cycle in Methanol-to-Olefins Reaction over ZSM-5 by Post-Synthetic Modification Using Calcium. *ChemCatChem,* **2016**, *8 (19)*, 3057–3063.

(2) Liutkova, A.; Zhang, H.; Simons, J. F. M.; Mezari, B.; Mirolo, M.; Garcia, G. A.; Hensen, E. J. M.; Kosinov, N. Ca Cations Impact the Local Environment inside HZSM-5 Pores during the Methanol-to-Hydrocarbons Reaction. *ACS Catal.,* **2023**, *13 (6)*, 3471–3484.

(3) Datka, J.; Turek, A. M.; Jehng, J. M.; Wachs, I. E. Acidic Properties of Supported Niobium Oxide Catalysts: An Infrared Spectroscopy Investigation. *J. Catal.,* **1992**, *135 (1)*, 186–199.

(4) Ishizuka, A.; Kimoto, K.; Ishizuka, K. Realtime Up-Sampling Noise Filter: Paradigm Shift for Data Acquisition. *Microsc. Microanal.,* **2020**, *26 (S2)*, 1936–1938.

(5) Jia, X.; Jiang, J.; Zou, S.; Han, L.; Zhu, H.; Zhang, Q.; Ma, Y.; Luo, P.; Wu, P.; Mayoral, A. Library Creation of Ultrasmall Multi-metallic Nanoparticles Confined in Mesoporous MFI Zeolites. *Angew. Chemie Int. Ed.,* **2021**, *60 (26)*, 14571–14577.

(6) Uslamin, E. A.; Kosinov, N.; Filonenko, G. A.; Mezari, B.; Pidko, E.; Hensen, E. J. M. Co-Aromatization of Furan and Methanol over ZSM-5—A Pathway to Bio-Aromatics. *ACS Catal.,* **2019**, *9 (9)*, 8547–8554.

(7) Welter, E.; Chernikov, R.; Herrmann, M.; Nemausat, R. A Beamline for Bulk Sample X-Ray Absorption Spectroscopy at the High Brilliance Storage Ring PETRA III. In *AIP Conf. Proceedings*, **2019**, *2054*, 40001–40005.

(8) Simonelli, L.; Marini, C.; Olszewski, W.; Avila Perez, M.; Ramanan, N.; Guilera, G.; Cuartero, V.; Klementiev, K. CLÆSS: The Hard X-Ray Absorption Beamline of the ALBA CELLS Synchrotron. *Cogent Phys.,* **2016**, *3 (1)*, 1–10.

(9) Kosinov, N.; Wijpkema, A. S. G.; Uslamin, E.; Rohling, R.; Coumans, F. J. A. G.; Mezari, B.; Parastaev, A.; Poryvaev, A. S.; Fedin, M. V; Pidko, E. A.; Hensen, E. J. M. Confined Carbon

Mediating Dehydroaromatization of Methane over Mo/ZSM-5. *Angew. Chem. Int. Ed. Engl.,* **2018**, *57* (4), 1016–1020.

(10) Ravel, B.; Newville, M. ATHENA, ARTEMIS, HEPHAESTUS: Data Analysis for X-Ray Absorption Spectroscopy Using IFEFFIT. *J. Synchrotron Radiat.,* **2005**, *12 (4)*, 537–541.

(11) Alain, M.; Jacques, M.; Diane, M.-B.; Karine, P. MAX: Multiplatform Applications for XAFS. In *J. of Physics: conference series*, **2009**, *190*, 12034.

(12) Liu, Y.; Ćoza, M.; Drozhzhin, V.; van den Bosch, Y.; Meng, L.; van de Poll, R.; Hensen, E. J. M.; Kosinov, N. Transition-Metal Catalysts for Methane Dehydroaromatization (Mo, Re, Fe): Activity, Stability, Active Sites, and Carbon Deposits. *ACS Catal.,* **2023**, *13 (1)*, 1–10.

(13) *mp-2472: SrO (Cubic, Fm-3m, 225)*. https://materialsproject.org/materials/mp-2472/ (accessed 2023-04-28).

(14) Clausen, B. S.; Steffensen, G.; Fabius, B.; Villadsen, J.; Feidenhans, R.; Topsøe, H. In Situ Cell for Combined XRD and On-Line Catalysis Tests: Studies of Cu-Based Water Gas Shift and Methanol Catalysts. *J. Catal.,* **1991**, *132 (2)*, 524–535.

(15) Liutkova, A.; Uslamin, E.; Parastaev, A.; Bolshakov, A.; Mezari, B.; Hensen, E. J. M.; Kosinov, N. A Scanning Pulse Reaction Technique for Transient Analysis of the Methanol-to-Hydrocarbons Reaction. *Catal. Today,* **2022**, *417 (113740)*, 1–8.

(16) Liu, Y.; Zhang, H.; Wijpkema, A. S. G.; Coumans, F. J. A. G.; Meng, L.; Uslamin, E. A.; Longo, A.; Hensen, E. J. M.; Kosinov, N. Understanding the Preparation and Reactivity of Mo/ZSM-5 Methane Dehydroaromatization Catalysts. *Chem. Eur. J.,* **2022**, *28 (5)*, 1–12.
